# Three new trixane glycosides obtained from the leaves of *Jungia sellowii* Less. using centrifugal partition chromatography

**DOI:** 10.3762/bjoc.12.68

**Published:** 2016-04-12

**Authors:** Luíse Azevedo, Larissa Faqueti, Marina Kritsanida, Antonia Efstathiou, Despina Smirlis, Gilberto C Franchi, Grégory Genta-Jouve, Sylvie Michel, Louis P Sandjo, Raphaël Grougnet, Maique Weber Biavatti

**Affiliations:** 1 Programa de Pós-Graduação em Farmácia, Universidade Federal de Santa Catarina, UFSC, Florianópolis, SC, Brazil; 2 Laboratoire de Pharmacognosie UMR/CNRS 8638 COMETE, Université Paris Descartes, Sorbonne Paris Cité, Faculté des Sciences Pharmaceutiques et Biologiques, 4 Avenue de l’observatoire 75006 Paris, France; 3 Laboratory of Molecular Parasitology, Department of Microbiology, Hellenic Pasteur Institute, 127 Vas. Sofias Ave, 11521 Athens, Greece; 4 Integrated Center for Childhood Onco-Hematological Investigation, State University of Campinas, P.O. Box 6141, 13083-970 Campinas, SP, Brazil

**Keywords:** CPC, guaiane, *Jungia*, trixanolide

## Abstract

*Jungia sellowii* (Asteraceae) is a shrub that grows in Southern Brazil and polar extract of its leaves presents anti-inflammatory properties. Cyperane, guaiane, nortrixane, and trixane sesquiterpene types were reported as the main metabolites in *Jungia* species. This work aims to describe the isolation and identification of sesquiterpenes in the leaves of *J. sellowii* using liquid–liquid partition and centrifugal partition chromatography. Thus, the crude extract of fresh leaves of *J. sellowii* was partitioned with hexane, dichloromethane, ethyl acetate and butanol, respectively. The butanol fraction was then subjected to a selected ternary system optimized for the CPC (centrifugal partition chromatography): ethyl acetate–ethanol–water (9:2:10, v/v/v). The separation was carried out isocratically at a flow rate of 25 mL/min at 1200 rpm, affording seven fractions A to G. TLC of fractions B, C and F displayed a single spot corresponding to three new glycosylated sesquiterpenoids. Their structures were established by using spectroscopic data in comparison to those reported in the literature. Furthermore, the isolates were evaluated for their leishmanicidal and cytotoxic effects. No cytotoxic effect was observed against the three cancer cell lines (HL60, JURKAT and REH), but compound **1** showed a weak antiprotozoal activity. Liquid–liquid partition and CPC turned to be a versatile technique of glycoside purification which is environmentally friendly and requires a limited amount of organic solvents.

## Introduction

*Jungia* (Asteraceae) comprises shrubs, lianas and herbs, widely distributed from Central to South America, including Southern Brazil. Species such as *J. paniculata* and *J. polita* are used in South America to disinfect and cure external wounds, to treat inflammation [1–2], and as a blood depurative [3]. Pharmacological studies demonstrated that the anti-inflammatory and antioxidant effects of *J. paniculata* were associated to the presence of flavonoids and other polyphenols [4]. Recently, we reported the in vivo anti-inflammatory properties of an aqueous fraction of the leaves of *J. sellowii*, that is in agreement with its popular use in Brazil [5].

Apart from the polyphenols identified in *Jungia* species, sesquiterpenoids with guaiane, guaiene, nortrixane, trixane (isocedrene), and cyperane scaffolds are also representative of this genus [6–9]. These terpenoids demonstrated a wide range of bioactivities [10–12], and hit compounds such as artemisinine, thapsigargin, and parthenolide are used nowadays for the treatment of malaria and cancer and have shown antileishmanial activities [13–14].

About two million new cases of *Leishmania* infection are considered to occur every year in tropical countries including Brazil. Today no effective vaccine for the prevention of *Leishmania* diseases exist, whereas current chemotherapy is ineffective due to the high toxicity, the emergence of drug resistance, and the high cost of treatment, among others [15–17]. Consequently, infected people betake of medicinal plants as an alternative to provide treatment.

Plants also have an important role as a source of antitumoral agents, and several anticancer drugs currently in use are derived from natural sources. Natural products often have selective biological actions due to binding affinities for specific proteins, and have superior chemical diversity and complexity, and frequently have more advantageous ADME/T properties [18].

Compared to other chromatographic methods, centrifugal partition chromatography (CPC) is compatible with green chemistry criteria since it does not use any polluting solid support such as silica. Moreover, it allows the complete recovery of the injected extract without degradation and only requires a limited amount of organic solvents [19], and it turned to be a versatile method of separation for the isolation of glycosides [20–21].

Based on the above observation, our aims were to identify new metabolites from *J. sellowii* and assess their antileishmanial and cytotoxic effects. To the best of our knowledge, there are no reports dealing with the isolation and structure characterization of glycosylated sesquiterpene derivatives from *Jungia sellowii*.

## Results and Discussion

### CPC separation

**Figure 1:**
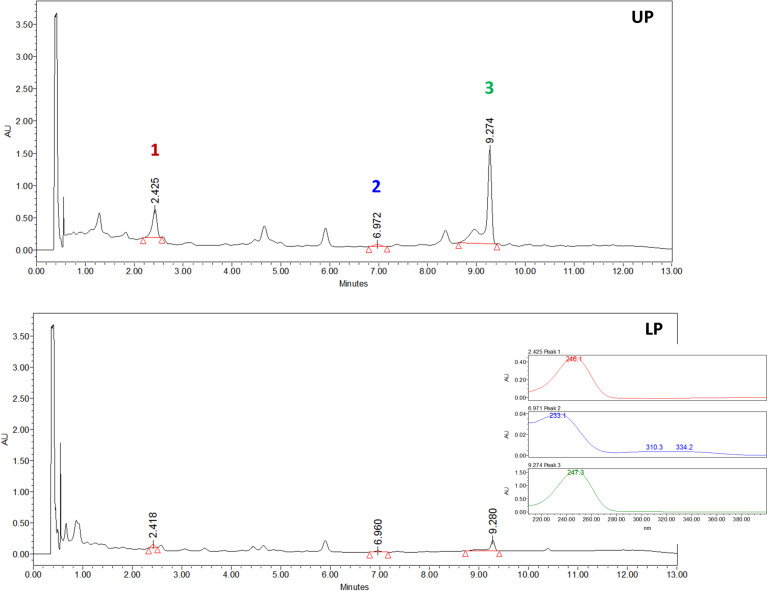
UPLC profile of the butanol fraction of the leaves of *Jungia sellowii* after shaking the flask with the selected biphasic system (details in the Experimental section). UP: upper phase (top chromatogram), LP: lower phase (bottom chromatogram). Detection at 242 nm.

**Figure 2:**
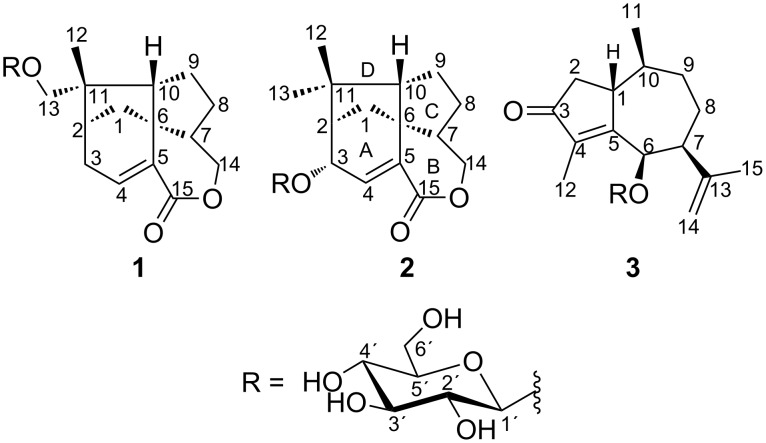
Structures of compounds **1**–**3**.

The aerial parts crude extract of *Jungia sellowii* was investigated using liquid–liquid partition and centrifugal partition chromatography (CPC), which is related to the counter-current chromatography (CCC) [22]. The chromatographic behavior of the butanol fraction of the leaves of *J. sellowii* was evaluated in six different biphasic systems consisting of different proportions of ethyl acetate (EtOAc)/ethanol (EtOH)/H_2_O by using the shake-flask method [23] (Table 1). These trials considered the performance of the phase’s separation and also the spots profile when monitored by TLC. Among them, the mixture of EtOAc/EtOH/H_2_O (9:2:10, v/v/v) gave a better separation, and it was used in the CPC equipment (details described in the experimental section) from which three new glycosylated sesquiterpenes were achieved in a single run in less than two hours (Figure 1). The compounds were identified to be two trixanolides and one guaianedienone (Figure 2) and the partition coefficient calculated for compounds **1** (16.20), **2** (2.77) and **3** (13.51) (Figure 1).

**Table 1:** Experimental conditions evaluated by using the shake-flask method.

Condition	Ethyl acetate	Ethanol	Water

1	9	2	10
2	8	2	10
3	7	2	10
4	9	1	10
5	9	3	10
6	8	3	10

Sesquiterpenes were previously reported from the genus *Jungia* [6–7,24–26]. Nevertheless, no glycosylated sesquiterpenes (and sesquiterpene lactones) were previously found in this genus. CPC has been used in a semi-empirical mode [20] as a replacement of vacuum liquid chromatography (VLC) or reversed-phase medium pressure liquid chromatography (MPLC), permitting specially the isolation of several terpene glycosides [27], such as a geranyl disaccharide [28], natural [29–30], semisynthetic iridoid derivatives [31], and diterpene glycosides [32]. This technique was for the first time used with *Jungia* extracts.

### Elucidation of the compounds

**Figure 3:**
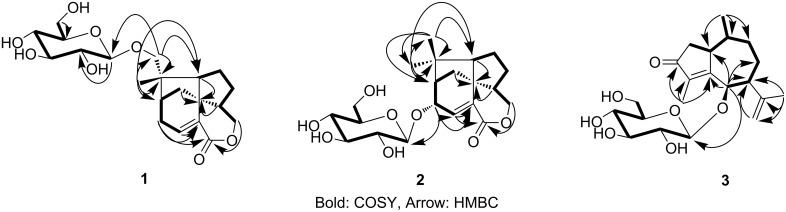
COSY and HMBC correlations of compounds **1**–**3**.

**Figure 4:**
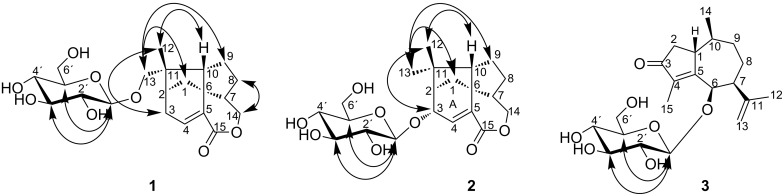
NOESY correlations of compounds **1**–**3**.

**Figure 5:**
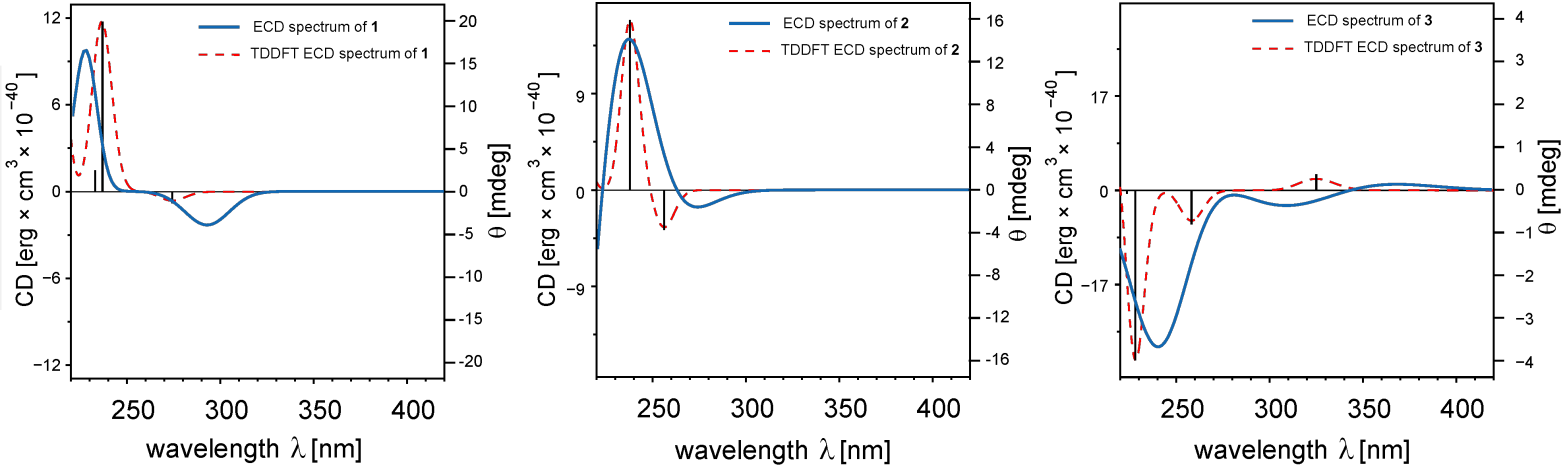
ECD spectra of compounds **1**–**3**.

Compound **1** was obtained as colorless gum. The molecular formula C_21_H_30_O_8_ was determined from its ESI–HRMS spectrum which gave the cationic ion peak [M + H]^+^ at *m*/*z* 411.1997 (calcd for 411.2019). The elemental composition indicated seven double bond equivalents. The ^13^C NMR spectrum of **1** displayed 21 signals: one CH_3_ group, seven CH_2_ groups, nine CH groups and four quaternary carbons. The study of the HSQC NMR correlation map revealed that among the CH_2_ groups, three were oxygenated while the CH groups included one anomeric (δ 4.28/104.7, Table 2 and Table 3), one olefin (δ 6.79/139.7), and four bearing oxygen (δ 3.20/75.0, 3.38/78.0, 3.32/71.8, and 3.30/77.4). A sugar moiety was deduced mainly from HSQC and COSY correlations observed between the oxymethine groups, the hemi-acetal and one of the CH_2_O (δ 3.65, 3.83/63.0) groups. Furthermore, the quaternary carbons included an α,β-unsaturated carbonyl (δ 165.5), an olefinic carbon (δ 137.5) and two sp^3^ carbons (δ 47.7 and 53.8). Fifteen carbon shifts remained after the sugar deduction suggesting the aglycone to be a sesquiterpene [33]. Based on previous reports, the signals of a CH group at δ 2.12/65.2 (C-10) and a quaternary carbon at δ 53.8 (C-6) observed in the NMR spectra of compound **1** suggested a trixane scaffold for this secondary metabolite [34]. C-10 and C-6 are respectively shared by two and four strained rings in the trixane skeleton, explaining their downfield resonances. COSY correlations (Figure 3) revealed from H-10 (δ 2.12) to H-9 (δ 1.63, 1.69), H-9 to H-8 (δ 1.58, 2.00), H-8 to H-7 (δ 2.11) which in turn correlated with H-14 (δ 4.04, 4.17) in addition to the HMBC correlations (Figure 3) from H-10 to C-6 (δ 53.8), H-7 to C-6, and H-14 to C-15 (δ 165.5) allowed to form the rings B and C. Moreover, C-6 had a long-range correlation with H-1 (δ 1.66, 2.07) which in turn displayed a COSY contact with H-2 (δ 2.38). This latter also correlated with H-3 (δ 2.44) which showed a similar interaction with H-4 (δ 6.79). The above correlations together with the long-range heteronuclear interactions between H-4 and C-6, as well as H-3, C-4 (δ 139.7) and C-5 (δ 137.5) allowed deducing the ring A. The last ring was established from the HMBC correlations observed between the protons of Me-12 and carbons C-2 (δ 43.0), C-10 (δ 65.2), C-11 (δ 47.7), and C-13 (δ 76.4). The sugar moiety was identified as glucopyranosyl by comparing its chemical shift with those formerly reported [35]. It was further attached to the aglycone at C-13 since H-13 (δ 3.39, 3.82) correlated with the anomeric carbon (104.7). NOE correlations (Figure 4) usually found in the β-D-glucopyranosyl core were also revealed between the anomeric proton H-1’ (δ 4.28), H-3’ (δ 3.38), and H-5’ (δ 3.30). The relative configuration of the aglycone was tentatively determined based on NOESY contacts observed between H-12, H-10, and H-3. Similarly, H-13 unveiled the same interactions with H-1 and H-9 while H-14 correlated with H-8 (Figure 4). In order to determine the absolute configuration of the compounds **1**–**3**, ECD spectra prediction was used [36–37]. The absolute configuration of **1** was assigned as 2*R*, 6*S*, 7*R*, 10*R*, and 11*S* by ECD analysis supported by the theoretical calculation using time-dependent density functional theory. Thus, two Cotton Effects (CE) from the n→π* transition of the α,β-unsaturated lactone were revealed at 225 and 275 nm with alternative signs (Figure 5). The aforementioned data in conjunction to the absolute configuration previously reported for trixanolides [34], led to identify compound **1** as a new member of the trixane sesquiterpenoids. The trivial name jungioside A was assigned.

**Table 2:** ^1^H NMR data [400 MHz, (CD_3_)_2_CO] of compounds **1**–**3**.

Position	Aglycone		

	**1**	**2**	**3**

1	1.66 (m), 2.07 (m)	1.72 (dd, 4.4, 11.5 Hz), 1.80 (br d, 11.5 Hz)	3.35 (m)
2	2.38 (m)	2.17 (m)	1.96 (m), 2.24 (dd, 7.2, 18.4)
3	2.44 (t, 3.6 Hz)	4.43 (dd, 2.1, 4.1 Hz)	–
4	6.79 (t, 3.6 Hz)	6.71 (dd, 1.5, 4.1 Hz)	–
5	–	–	–
6	–	–	4.93 (br s)
7	2.11 (m)	2.14 (m)	1.97 (m)
8	1.58 (m), 2.00 (m)	1.71 (m), 2.05 (m)	1.09 (m), 1.30 (m)
9	1.63 (m), 1.69 (m)	1.56 (m), 1.67 (m)	1.57 (m), 2.02 (m)
10	2.12 (overlapped)	1.90 (m)	2.51 (m)
11	–	–	–
12	1.12 (s)	1.05 (s)	1.91 (br s)
13	3.39 (d, 9.2 Hz), 3.82 (d, 9.2 Hz)	1.10 (s)	4.82 (m), 4.97 (br s)
14	4.04 (dd, 5.5, 11.7 Hz), 4.17 (dd, 4.3, 11.7 Hz)	4.11 (dd, 4.9, 11.7 Hz), 4.25 (dd, 4.2, 11.7 Hz)	0.93 (d, 7.2 Hz)
15	–	–	1.66 (br d, 2.2 Hz)
Glucopyranosyl			
1´	4.28 (d, 7.7 Hz)	4.47 (d, 7.7 Hz)	4.39 (d, 7.8 Hz)
2´	3.20 (*pseudo*-t, 8.3 Hz)	3.16 (dd, 7.7, 8.8 Hz)	3.27 (m)
3´	3.38 (m)	3.38 (m)	3.24 (m)
4´	3.32 (m)	3.33 (m)	3.33 (m)
5´	3.30 (m)	3.33 (m)	3.37 (m)
6´	3.65 (dd, 5.0, 11.5 Hz), 3.83 (m)	3.66 (dd, 4.9, 11.7 Hz), 3.83 (dd, 7.8, 11.7 Hz)	3.55 (dd, 5.5, 11.7 Hz) 3.69 (dd, 3.1, 11.7 Hz)

**Table 3:** ^13^C NMR data [100 MHz, (CD_3_)_2_CO] of compounds **1**–**3**.

Position	Aglycone		

	**1**	**2**	**3**

1	39.5	35.7	44.6
2	43.0	53.4	36.8
3	32.3	74.9	208.5
4	139.7	137.3	134.0
5	137.5	139.5	177.8
6	53.8	54.7	80.9
7	40.3	40.5	52.3
8	31.0	31.5	30.1
9	25.9	27.0	29.2
10	65.2	63.2	33.2
11	47.7	40.1	149.3
12	24.7	28.7	22.9
13	76.4	28.4	110.9
14	68.7	68.8	20.3
15	165.5	165.6	7.4
Glucopyranosyl			
1´	104.7	103.6	104.8
2´	75.0	74.9	74.8
3´	78.0	78.0	77.1
4´	71.8	71.7	70.9
5´	77.4	77.6	76.5
6´	63.0	63.0	62.1

Compound **2** was obtained as a colourless gum. The molecular formula C_21_H_30_O_8_ was determined from its ESI–HRMS which gave the cationic ion peak [M + H]^+^ at *m*/*z* 411.2013 (calcd for 411.2013). The elemental composition was consistent with seven double bond equivalents. The NMR spectra of compound **2** displayed similar features as compound **1** except for the presence of one more CH_3_ group, an additional CH group and the absence of two CH_2_ groups. The presence of resonances at δ 54.7 (C-6) and δ 1.90/63.2 (H-10/C-10) suggested **2** to be also a trixane-type as its congener **1** [1–2]*.* The sugar moiety was attached at C-3 based on COSY correlation (Figure 3) of H-4 (δ 6.71) and H-3 (δ 4.43) in addition to the long-range correlation observed in the HMBC spectrum (Figure 3) from H-3 to the anomeric carbon (δ 103.6). The coupling constant of the anomeric proton (*J* = 7.7 Hz) and the NOESY correlation of this latter with H-3´ (δ 3.38) and H-5´ (δ 3.33) were consistent with β-D-glucopyranosyl moiety [38]. Besides, the stereochemistry of chiral centers in the aglycone was tentatively assigned as those previously reported for the trixane skeleton. Relative configurations of compound **2** turned to be similar as that of compound **1** since H-12 (δ 1.05) revealed NOE contact (Figure 4) with H-3 (δ 4.43) and H-10 (δ 1.90) likewise, H-13 (δ 1.10) showed similar interactions with H-1 (δ 1.72) and H-9 (δ 1.56, 1.67).

The absolute configuration of **2** was determined to be 2*R*, 3*S*, 6*S*, 7*R*, and 10*S* by ECD analysis. As compound **1**, two Cotton Effects (CE) from the n→π* transition of the α,β-unsaturated lactone were revealed at 225 and 275 nm with alternative signs (Figure 5). The complete assignment in conjunction to the data found in the literature led to identify compound **2** as new trixane congener (Figure 2). The trivial name jungioside B was given.

The NMR data of **3** revealed signals of a β-D-glucopyranosyl (δ 4.39/104.8, 3.27/74.8, 3.24/77.1, 3.33/70.9, 3.37/76.5, 3.55 and 3.69/62.1) as found in the above-mentioned compounds and signals of fifteen carbons suggesting another sesquiterpene bearing a sugar moiety. The diagnostic of 2D experiments permitted to identify the aglycone as a guaiane-type sesquiterpene whose the structure was consistent with 6-hydroxyguaiane previously reported [39]. Moreover, the HMBC correlation (Figure 3) from the hydrogen at δ 4.93 to the anomeric carbon (δ 104.8) revealed the presence of an osidic bond. Thus, the molecular formula C_21_H_32_O_7_ was deduced from the aforementioned information in conjunction to the ESI–HRMS which gave the pseudo-molecular ion peak at *m*/*z* 397.2239 [M + H]^+^ (calcd. 397.2226). The elemental composition corresponded to six double bond equivalents. Some spatial correlations were observed in the NOESY spectrum from H-1 to H-14 and from H-6 to H-7 consistent with the relative configuration reported the aglycone [39]. The absolute configuration of **3** was assigned as 1*R*, 6*R*, 7*S*, and 10*S*. Three CE's were observed on the experimental ECD spectrum at 240, 305 and 350 nm due to the n→π* transition of the α,β-unsaturated ketone. The foregoing data led to identify **3** as 6-hydroxyguaiane congener (Figure 2) and the trivial name guaianoside was given.

Trixane derivatives have recently been reported as antileishmanial metabolites [40], and on the basis of these results the butanol fraction from the areal parts crude extract as well as its isolated compounds (**1**–**3**) were evaluated for their leishmanicidal activity against *L. donovani*, *L. infantum* and *L. amazonensis* (promastigotes and intracellular amastigotes). The cytotoxicity of the butanol fraction in murine macrophages was found weak, with an IC_50_ value at 290 μg/mL. However, the butanol fraction displayed activity against *L. amazonensis* intracellular amastigotes with an IC_50_ value of 100 μg/mL. Except for compound **1** that exhibited a weak antileishmanial activity at 50 μM (20%) against *L. amazonensis* intracellular amastigotes, none of the other sesquiterpenes displayed antiparasitic activity.

Compounds **1**–**3** showed less than 50% antiproliferative effect on leukemic cell lines HL60, JURKAT and REH at 15 μM.

## Conclusion

The chemical study of the leaves of *J. sellowii* led to the isolation and characterization of three new sesquiterpene glycosides, including the first report of trixane lactone glycosides. Liquid–liquid partition and CPC proved to be a very useful technique for the investigation of polar extracts. Moreover, the commercial availability of industrial instruments enables scale-up to batch production for high-scale isolation. The CPC technique turned to be a versatile analytical tool leading to the purification and identification of new glycosylated sesquiterpenes including a rare skeleton (trixane). Only few of them were described from the genus *Jungia* along with polyacetylenes, coumarins and flavonoids [6–7,24–26]. Globally, the tested glycosylated sesquiterpenes displayed no or weak activity against *Leishmania* strains, and displayed no cytotoxicity against murine macrophages and the leukemic cancer cell lines.

## Experimental

### Solvents, materials and instruments

Ethanol for extraction and organic solvents for partitioning (hexane, dichloromethane, ethyl acetate, and butanol) as well as for CPC were pro-analysis grade (p.a.). Water was distilled from deionized water whereas MeOH and acetonitrile for UHPLC were of analytical grade (HPLC grade).

Merck precoated silica gel 60 F_254_ plates, 0.25 mm thickness, were used for analytical thin-layer chromatography. The visualization of spots on TLC plates was effected by exposure to UV 254 nm and by spraying with sulfuric vanillin solution at 30% and heating. The mobile phase used to monitor the method development and the fractions was composed by EtOAc/CH_2_O_2_/AcOH/H_2_O (60:0.6:0.6:20). The 1D and 2D NMR experiments were recorded with Bruker AC-300 and Bruker Avance-400 spectrometers at 400 MHz for ^1^H and 2D NMR and at 75 MHz for ^13^C NMR. The spectra were recorded using deuterated solvents CDCl_3_ and CD_3_OD. Chemical shifts (δ) are expressed in ppm with reference to the TMS signal (δ_H_/δ_C_ 0.0) and coupling constants are reported in Hz. The 2D NMR experiments (COSY, HSQC, HMBC, and NOESY) were performed using standard Bruker microprograms. UPLC-PDA analyses were performed on a Waters Acquity H UPLC quaternary system manager equipped with a Acquity sample manager and a PDA detector. Data were processed with Empower 3 software. CPC separation was performed on a SCPC-250+1000-B apparatus provided by Armen Instrument (Saint-Avé, France) fitted with a 1000 mL rotor containing 2016 twin-cells, equipped with a gradient pump and a 50 mL loop injection 6-way valve.

Electronic circular dichroism (ECD) spectra were recorded in acetonitrile using a Jasco XLC 3195CD detector.

### Plant material

The leaves of *J. sellowii* Less. were collected in Rio Negrinho, Santa Catarina, Brazil, in March 2012. Plant identification was performed by the botanist Dr. Ademir Reis from the botany department at the Federal University of Santa Catarina, and a voucher specimen (RB number 537.991) is preserved in the Jardim Botanico do Rio de Janeiro, Brazil.

### Measurement of the partition coefficient (*K* value)

First, the selected solvent system (EtOAc/EtOH/H_2_O, 9:2:10, v/v/v) was prepared and equilibrated, then 1 mL of the upper phase and 1 mL of the lower phase were taken to a test tube and about 1 mg sample was added into it. The test tube was shaken vigorously and allowed to settle for 5 min. About 0.5 mL of upper and lower phases were taken into two vials and evaporated under nitrogen. The residues of each phase were dissolved in 1 mL of methanol and were then analyzed by UPLC (Figure 1). The *K* value was expressed as the peak area of compounds in upper phase divided by that in the lower phase.

Chromatographic conditions employed for the peak area measurement were column Acquity UPLC BEH C18 1.7 µm (2.1 × 50 mm), with a flow rate of 0.3 mL/min using gradient mode composed of formic acid 0.1% (A) and acetonitrile (B): starting 85% of A, changing to 82% of A in 3 min, to 78% of A in 5 min, to 65% of A in 10 min, returning to the initial conditions in 12 min. The detection was done at a wave length of 242 nm.

### Computational details (ECD)

All calculations were conducted using Gaussian 09W [41]. After geometry optimization using density functional theory (DFT) at the B3LYP/6-311+g (d,p) level of theory. A check for imaginary frequencies was performed in order to confirm the presence of a real minimum. Calculations of the rotational strengths and excitation energies were realized using time dependent (TD) DFT at the same level of theory. ECD spectra were plotted using the SpecDis v1.61 software [42].

### Extraction, fractionation and isolation procedure

Fresh leaves of *J. sellowii* (1.8 kg) were macerated in 3 L of EtOH/H_2_O (1:1) that after solvent removal furnished 40 g of crude extract. This crude extract was dissolved in 600 mL of cold water and partitioned with solvents of increasing polarity, giving hexane (hex, 0.4 g), dichloromethane (DCM, 1 g), EA (0.6 g) and BuOH (4.6 g) fractions, together with the remaining aqueous fraction (lyophilized, 31.9 g). CPC separation of the BuOH fraction (4 g) was carried out in the optimized biphasic system composed by EtOAc/EtOH/H_2_O, 9:2:10, v/v/v, shaken in a separatory funnel and kept until the phase separation.

The separation was then conducted in isocratic ascending mode at room temperature, using the lower phase of the previously prepared mixture as stationary phase and the upper phase as mobile phase. The 1 L column was first filled with the lower phase in ascending mode at a flow-rate of 50 mL/min at 500 rpm. Rotation speed was then set up at 1200 rpm and the mobile phase pumped through the stationary phase at a flow-rate of 25 mL/min until equilibration. The retention volume was determined as 320 mL.

The butanol fraction was injected after dissolution in 20 mL of a mixture 1:1 of the selected biphasic system. 70 fractions of 50 mL were collected in the ascending mode. After switching to descending mode, 20 additional fractions of 50 mL were collected. Extrusion process started after finishing collection of the tubes by pumping the stationary phase into the column at a ﬂow rate of 25 mL/min. Fractions collected in the descending mode (tubes 70–90) did not lead to any interesting outcome. However, Fractions from the ascending mode were pooled together based on their TLC profile affording fractions A–G. Fractions B (96 mg), C (85 mg) and F (90 mg) provided a single spot on the TLC plate, resulting in the elucidated new glycosylated sesquiterpenoids **3**, **2** and **1**, respectively (Figure 2).

Compound **1**: colourless gum; IR λ_max_ (cm^−1^): 3390.8, 2924.2, 1705.7, 1627.9, 1420.8, 1363.4, 1265.9, 1221.8, 1162.1, 1077.4, 1035.0; ESI–HRMS *m*/*z* 411.1997 [C_21_H_30_O_8_ + H]^+^ (calcd. 411.2019), 821.3940 [2M + H]^+^. For ^1^H and ^13^C NMR data see Table 2 and Table 3.

Compound **2**: colourless gum; IR λ_max_ (cm^−1^): 3396.4, 2924.4, 2872.4, 1704.7, 1458.8, 1364.4, 1266.2, 1221.3, 1074.8, 1036.4; ESI–HRMS *m*/*z* 411.2013 [C_21_H_30_O_8_ + H]^+^ (calcd. 411.2019), 433.1839 [M + Na]^+^. For ^1^H and ^13^C NMR data see Table 2 and Table 3.

Compound **3**: colourless gum; IR λ_max_ (cm^−1^): 3376.4, 2938.0, 1688.2, 1080.5; ESI–HRMS *m*/*z* 397.2239 [C_21_H_32_O_7_ + H]^+^ (calcd. 397.2226), 793.4391 [2M + H]^+^. For ^1^H and ^13^C NMR data see Table 2 and Table 3.

### Bioactivity tests

#### Evaluation of antileishmanial activity

*L. donovani* (strain LG13, MHOM/ET/0000/HUSSEN), *L. infantum* (MHOM/GR/2002/GH12) and *L. amazonensis* (MPRO/BR/72/M1845) promastigotes and the murine macrophage J774 cell line (American Type Culture Collection, Manassas, VA) were cultured in RPMI 1640 (RPMI) medium, respectively, supplemented with 10% heat-inactivated fetal bovine serum, 10 mM HEPES and antibiotics (penicillin/streptomycin) as previously described at 26 C [43]. The inhibitory activity of compounds was determined with the use of an MTT-based assay, the Alamar blue, as previously described [44]. The 50% maximal inhibitory concentration (IC_50_) was calculated using a nonlinear regression curve fit [45].

For evaluating the inhibitory activity of compounds against intracellular amastigotes, J774 macrophages were seeded into 96-well flat bottom plates at a density of 2 × 10^5^ cells/mL in 200 μL RPMI and were left to adhere overnight at 37 °C in 5% CO_2_. 24 h later macrophages were infected with stationary phase promastigotes at a ratio of 10 parasites per macrophage and incubated for a further 24 h at 37 °C in 5% CO_2_ as previously described [44].

All experiments were performed at least three independent times in triplicate.

#### In vitro cytotoxicity against leukemic cells

The cell lines used in this study were HL60 (Acute Promyelocytic Leukemia), JURKAT (Acute T cell Leukemia) and REH (Acute Lymphocytic Leukemia non-T; non-B). The cells were grown in plastic bottles (75 cm^3^) containing RPMI 1640 (Sigma R6504) medium supplemented with 10% fetal calf serum (Gibco 16000-044), 1% penicillin (10000 IU/mL), and streptomycin (10 mg/mL) (Gibco 15070) at 37 °C in humidified air with 5% CO_2_. The medium was changed every 48 h.

The cytotoxicity of each compound in the cell lines indicated above was determined by the MTT (3-(4,5-dimethylthiazol-2-yl)-2-5-diphenyltetrazolium bromide) assay [46]. The absorbance was read in a Synergy ELISA plate reader (Bio Tek Instruments, Highland Park, Winooski, USA) at 570 nm. The results were expressed as percentage inhibition relative to control cells (considered as 100%).

## Supporting Information

File 1 NMR and MS spectra of compounds **1**–**3**.
