# Supplementary material for: Three new trixane glycosides obtained from the leaves of Jungia sellowii Less. using centrifugal partition chromatography
Source: Beilstein J Org Chem. 2016 Apr 12;12:674–83. doi: 10.3762/bjoc.12.68 (PMC4902082; doi:10.3762/bjoc.12.68)

Supporing Information

for

Three new trixane glycosides obtained from the leaves of *Jungia sellowii* Less. using centrifugal partition chromatography

Luíse Azevedo[1], Larissa Faqueti[1], Marina Kritsanida[2], Antonia Efstathiou[3], Despina Smirlis[3], Gilberto C. Franchi Jr[4] ,Grégory Genta-Jouve[2], Sylvie Michel[2], Louis P. Sandjo[1], Raphaël Grougnet[*,§,2] and Maique W. Biavatti*[,¶,1].

Address: [1]Programa de Pós-Graduação em Farmácia, Universidade Federal de Santa Catarina – UFSC, Florianópolis, SC – Brazil, [2]Laboratoire de Pharmacognosie UMR/CNRS 8638 COMETE, Université Paris Descartes, Sorbonne Paris Cité, Faculté des Sciences Pharmaceutiques et Biologiques, 4 Avenue de l'observatoire 75006 Paris, France, [3]Laboratory of Molecular Parasitology, Department of Microbiology, Hellenic Pasteur Institute, 127 Vas. Sofias Ave, 11521 Athens, Greece and [4]Integrated Center for Childhood Onco-Hematological Investigation, State University of Campinas, P.O. Box 6141, 13083-970 Campinas, SP, Brazil

Email: Maique W. Biavatti - maique.biavatti@ufsc.br; Raphaël Grougnet - raphael.grougnet@parisdescartes.fr

*Corresponding author

[§]Phone number: +33 1 53 73 9806.

[¶]Phone number: +55 48 3721 3493

NMR and MS spectra of compounds 1–3

1. ^{1}H NMR spectrum of the sesquiterpene lactone **1** (400 MHz, acetone-d_6).

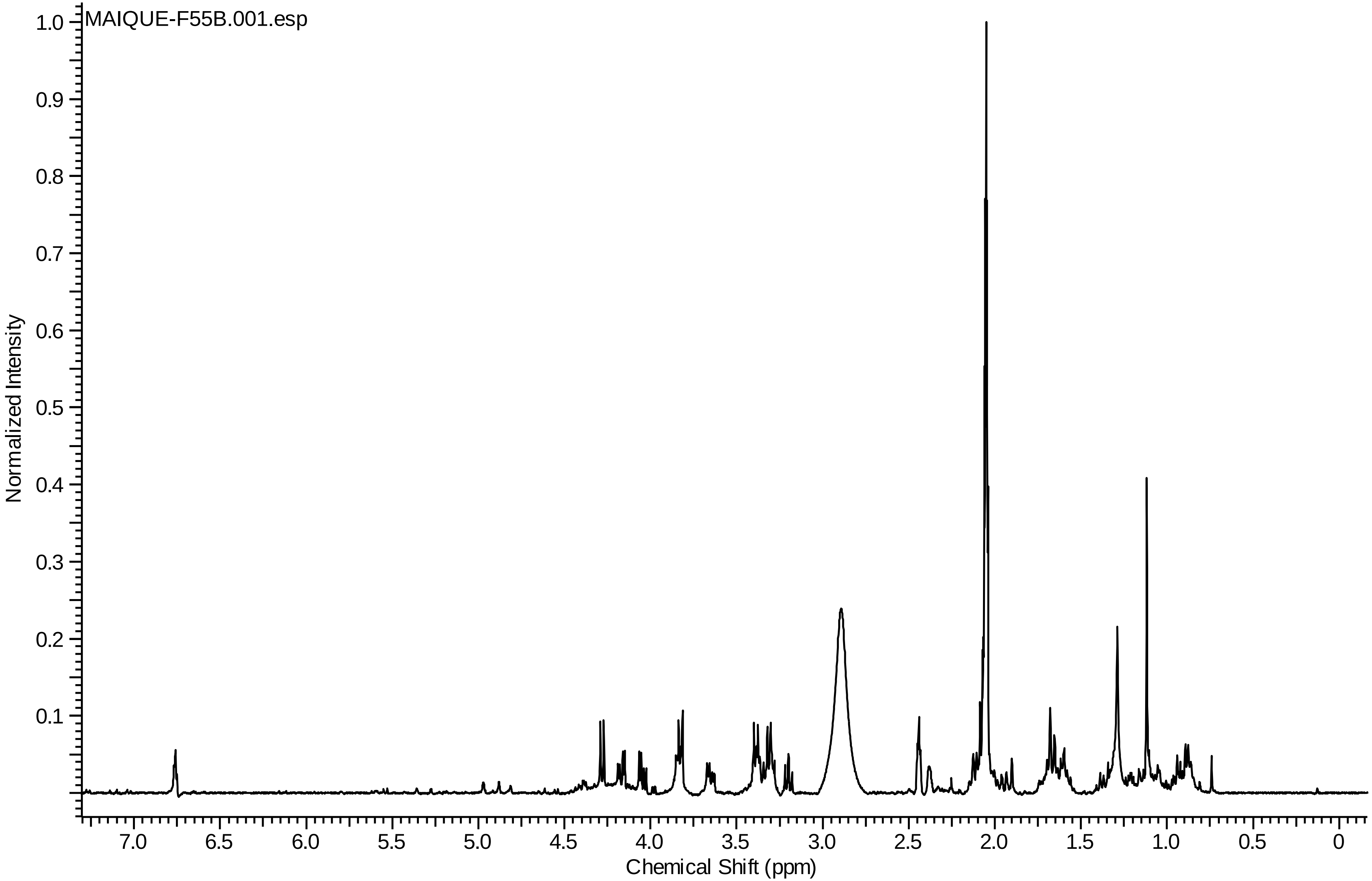

2. ^{13}C NMR spectrum of the sesquiterpene lactone **1** (100 MHz, acetone-d_6).

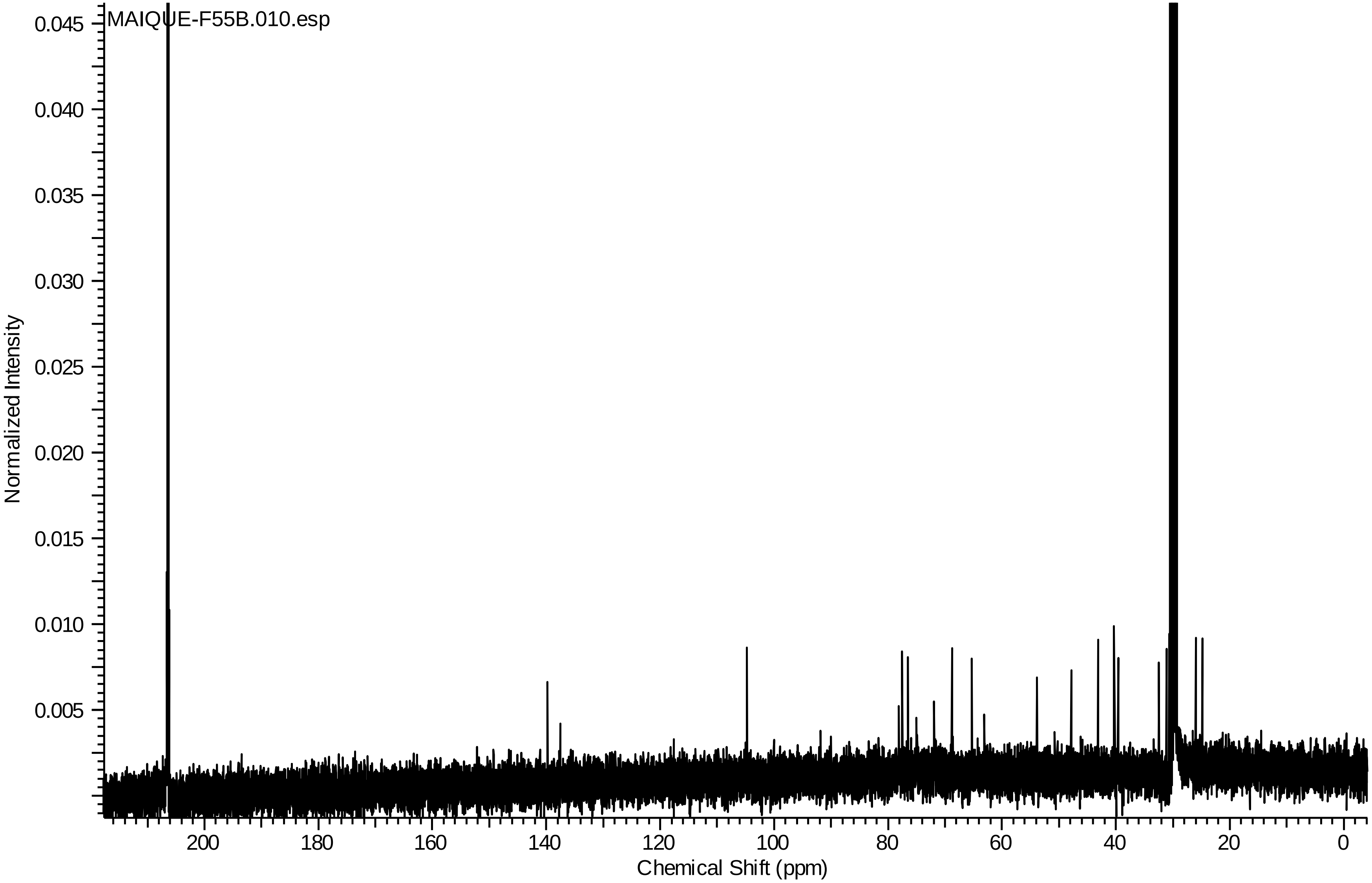

3. 2D NMR HSQC correlation map of the sesquiterpene lactone **1** (100/400 MHz, acetone- d_6).

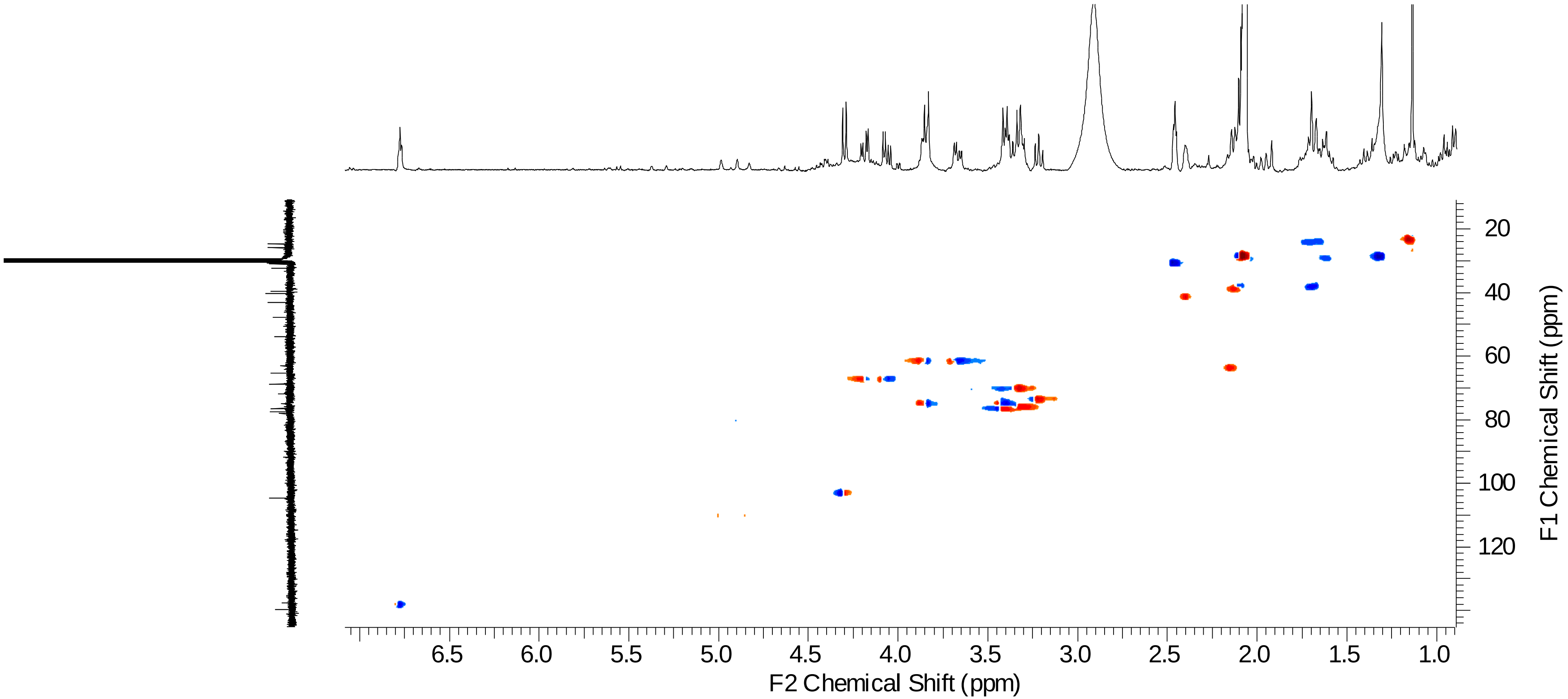

4. 2D NMR COSY correlation map of the sesquiterpene lactone **1** (400/400 MHz, acetone- d_6).

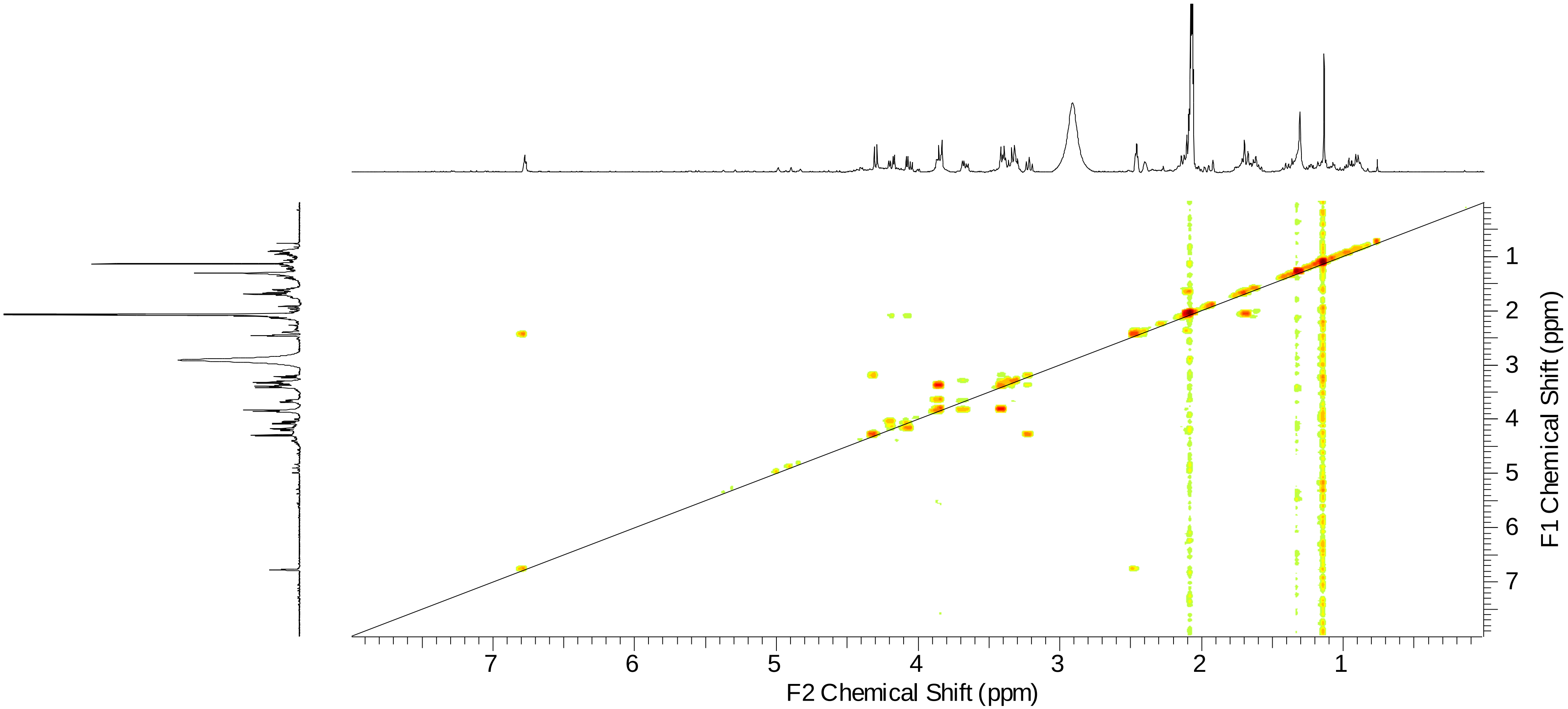

5. 2D NMR HMBC correlation map of the sesquiterpene lactone **1** (100/400 MHz, acetone- d_6).

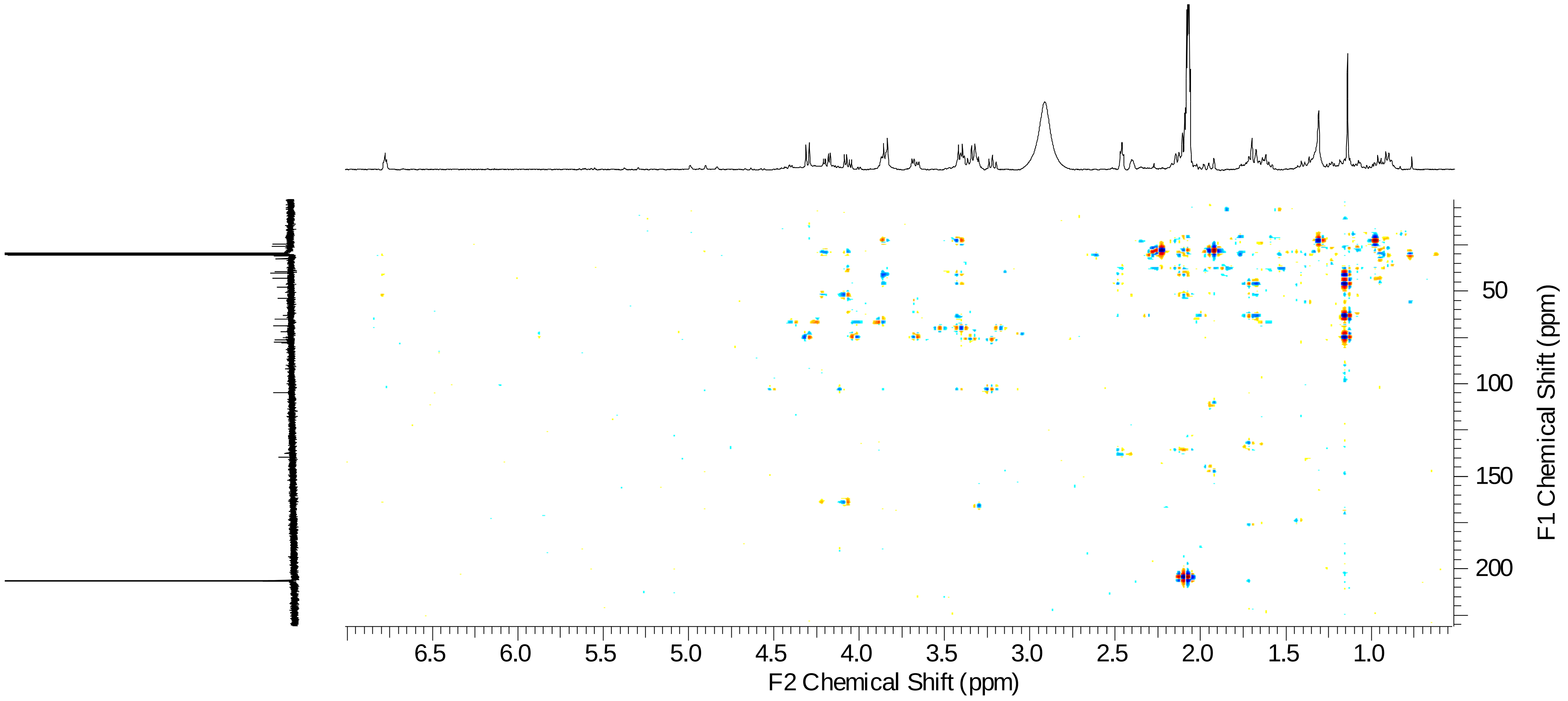

6 2D NMR NOESY correlation map of the sesquiterpene lactone **1** (400/400 MHz, acetone- d_6).

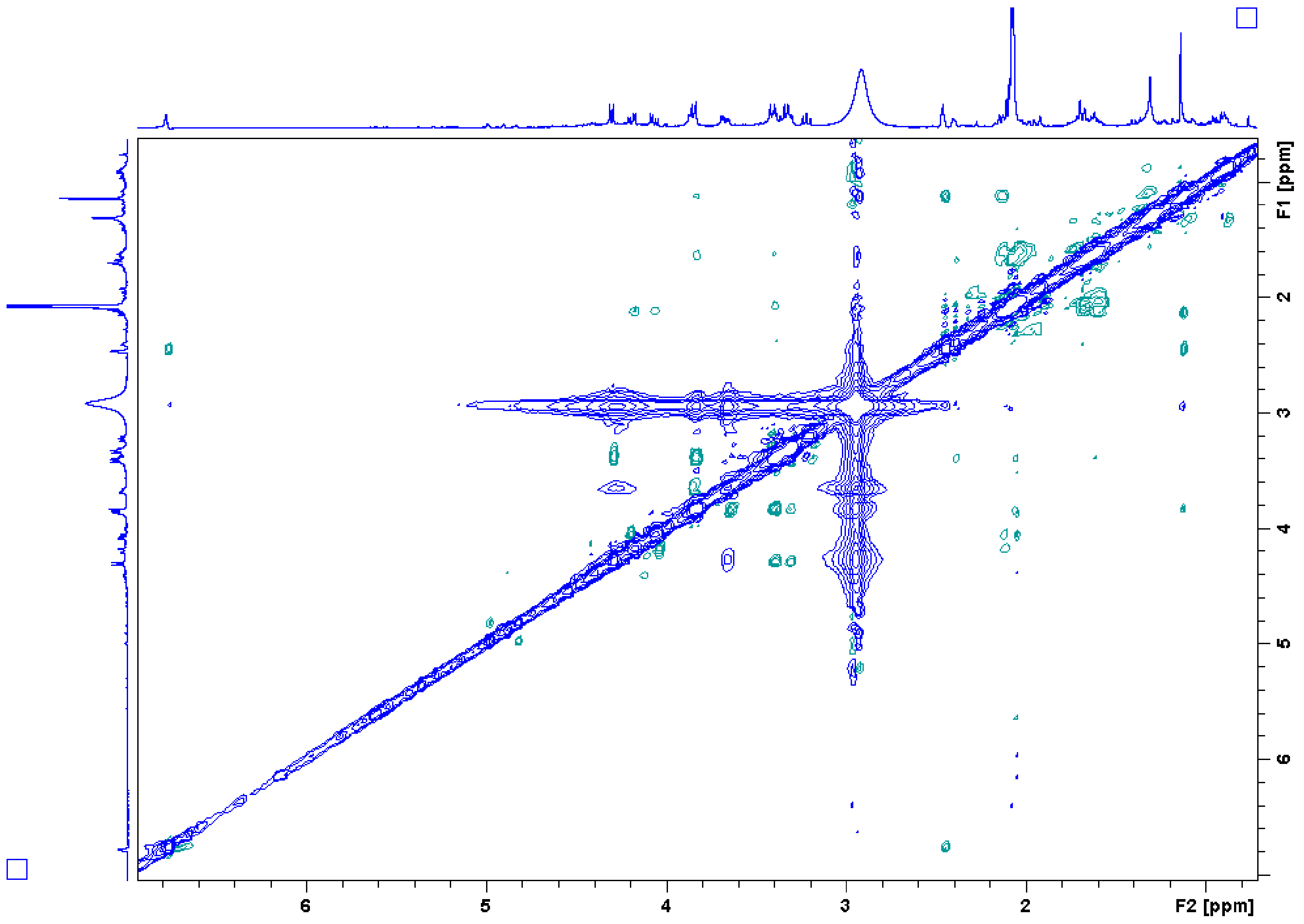

7 HR-MS spectrum of the sesquiterpene lactone **1**

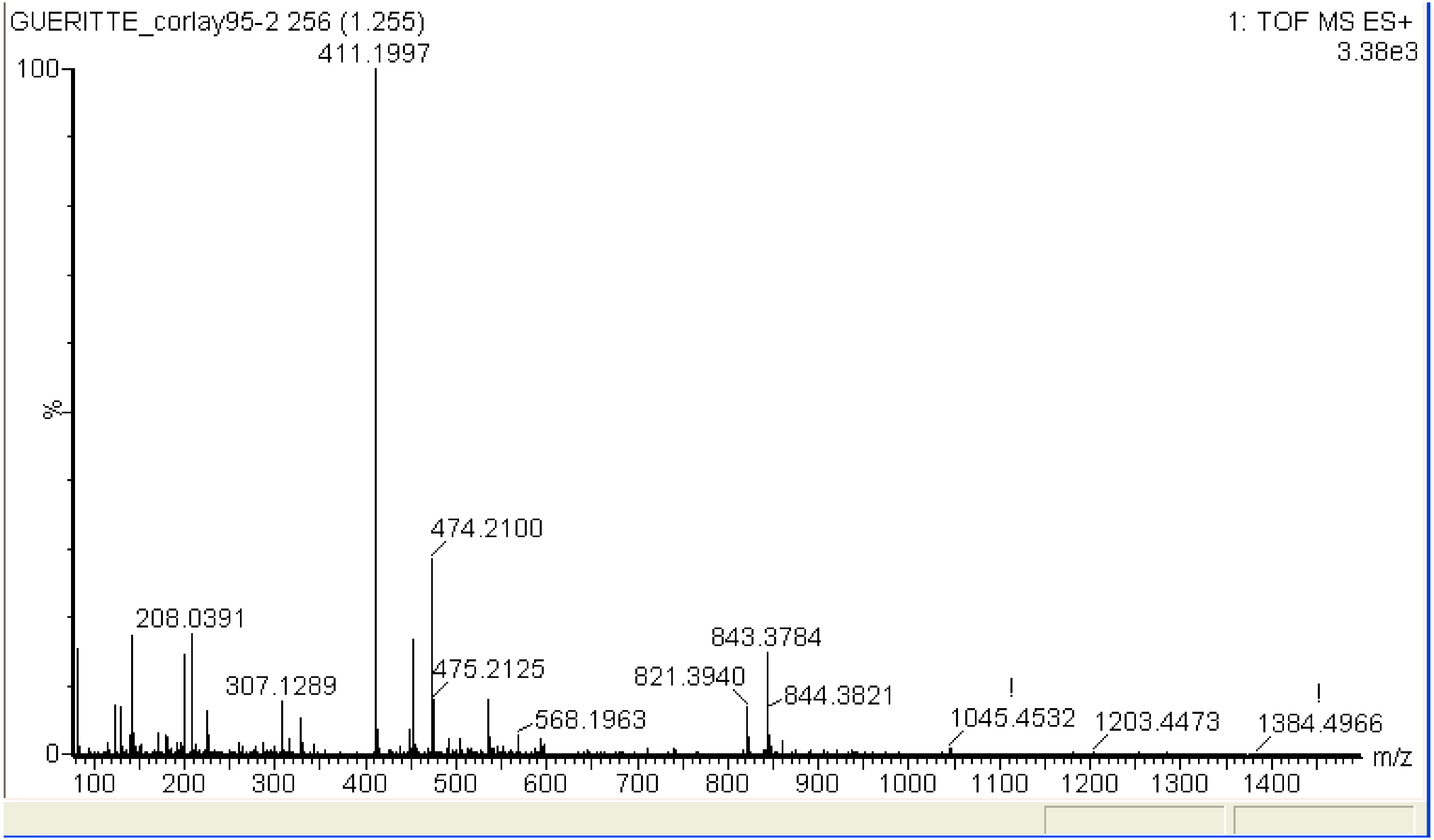

8 ^{1}H NMR spectrum of the sesquiterpene lactone **2** (400 MHz, acetone- d_6).

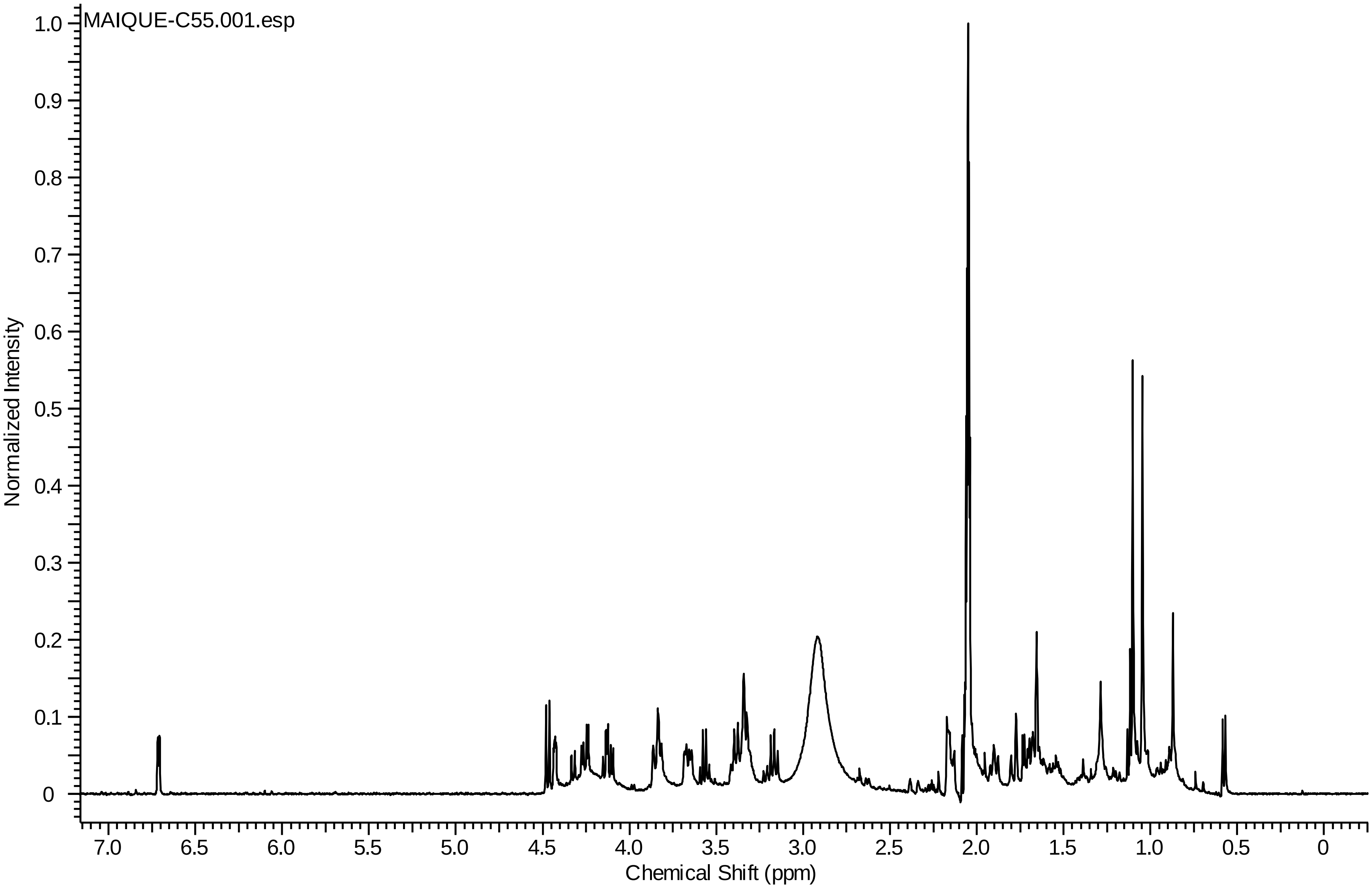

9 ^{13}C NMR spectrum of the sesquiterpene lactone **2** (100 MHz, acetone- d_6).

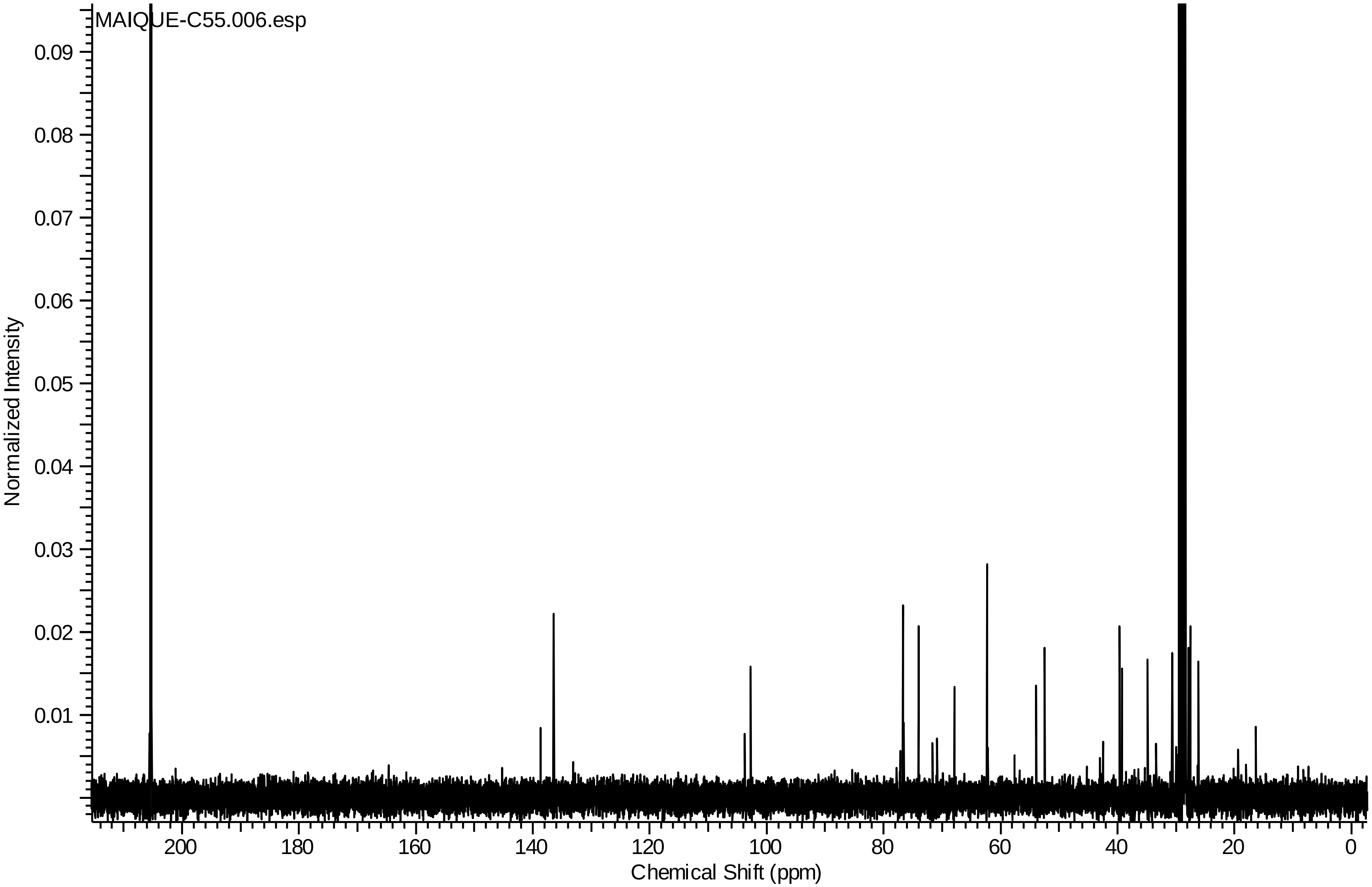

10 2D NMR HSQC correlation map of the sesquiterpene lactone **2** (100/400 MHz, acetone- d_6).

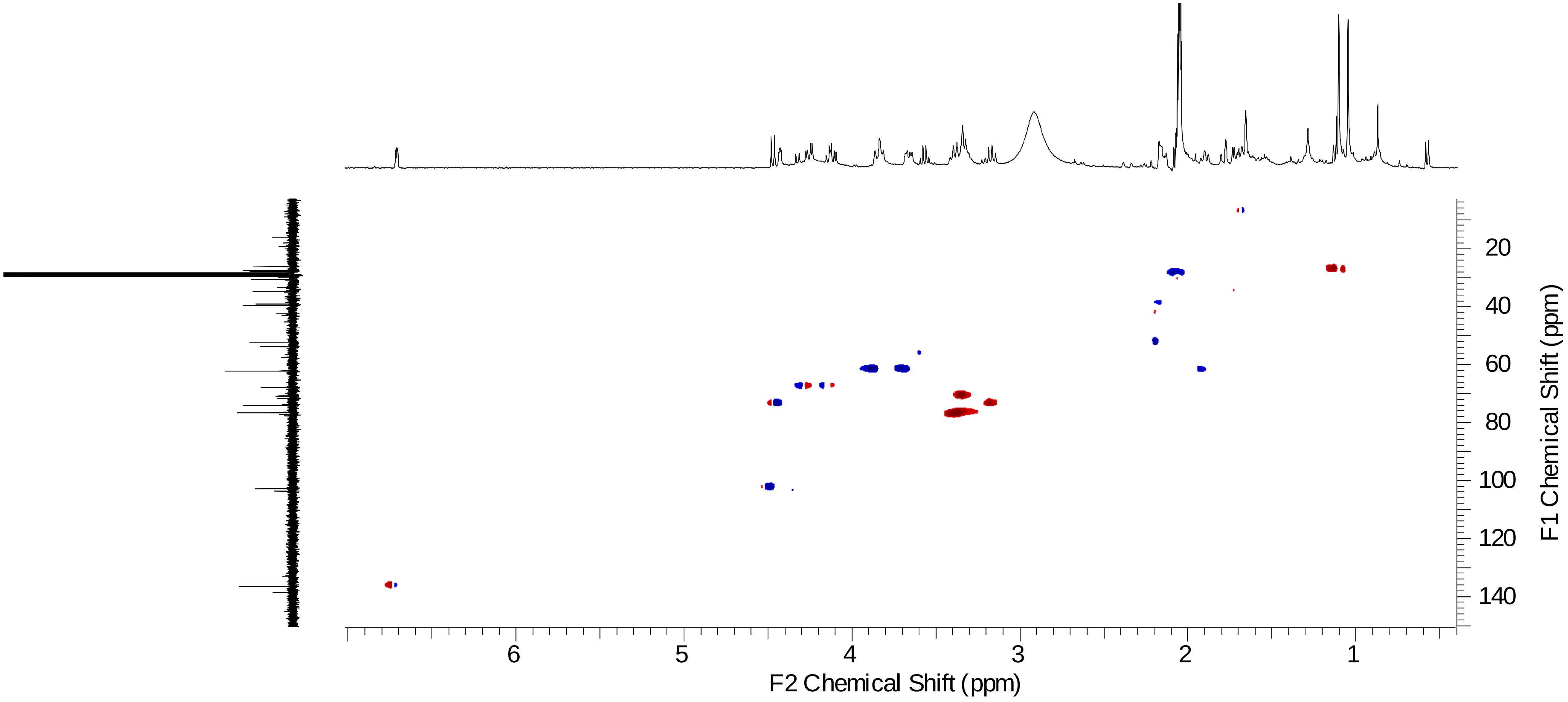

11 2D NMR COSY correlation map of the sesquiterpene lactone **2** (400/400 MHz, acetone- d_6).

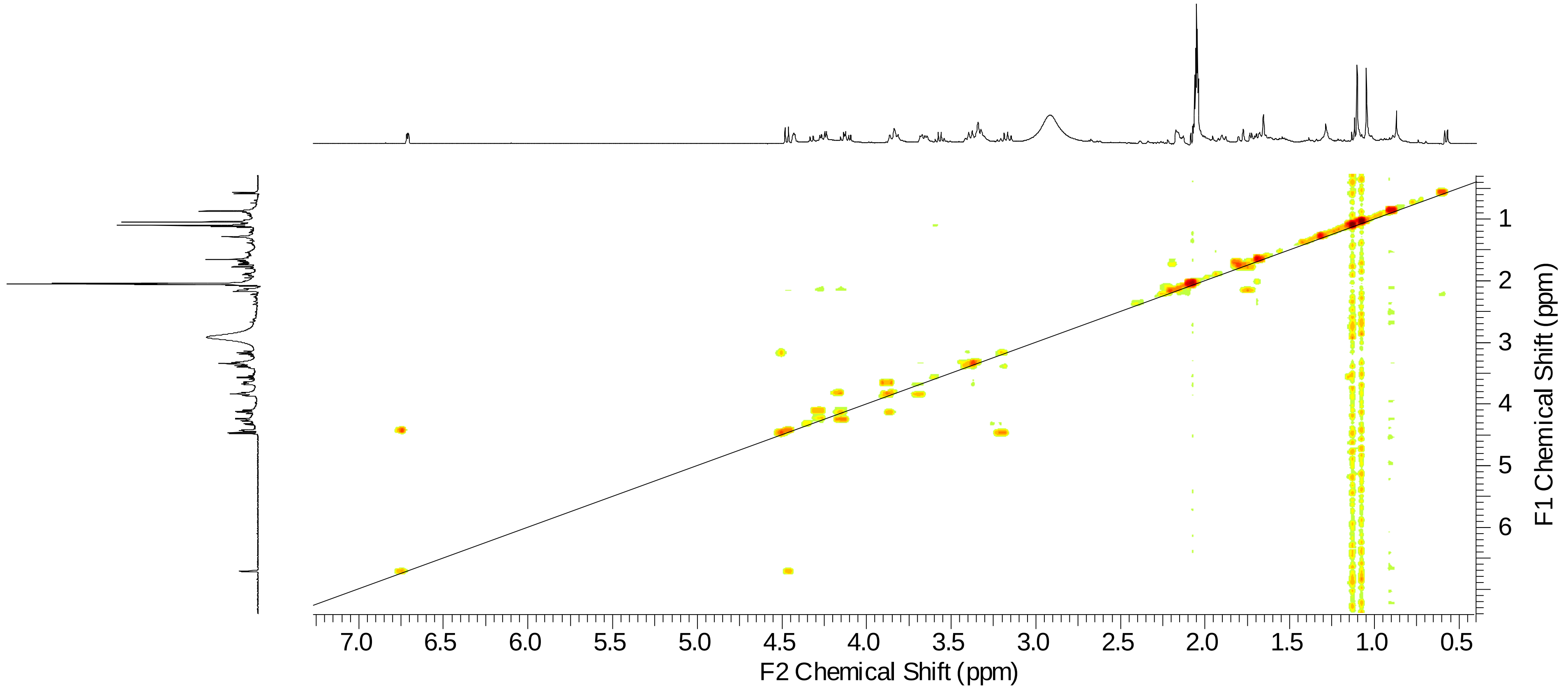

12 2D NMR HMBC correlation map of the sesquiterpene lactone **2** (100/400 MHz, acetone- d_6).

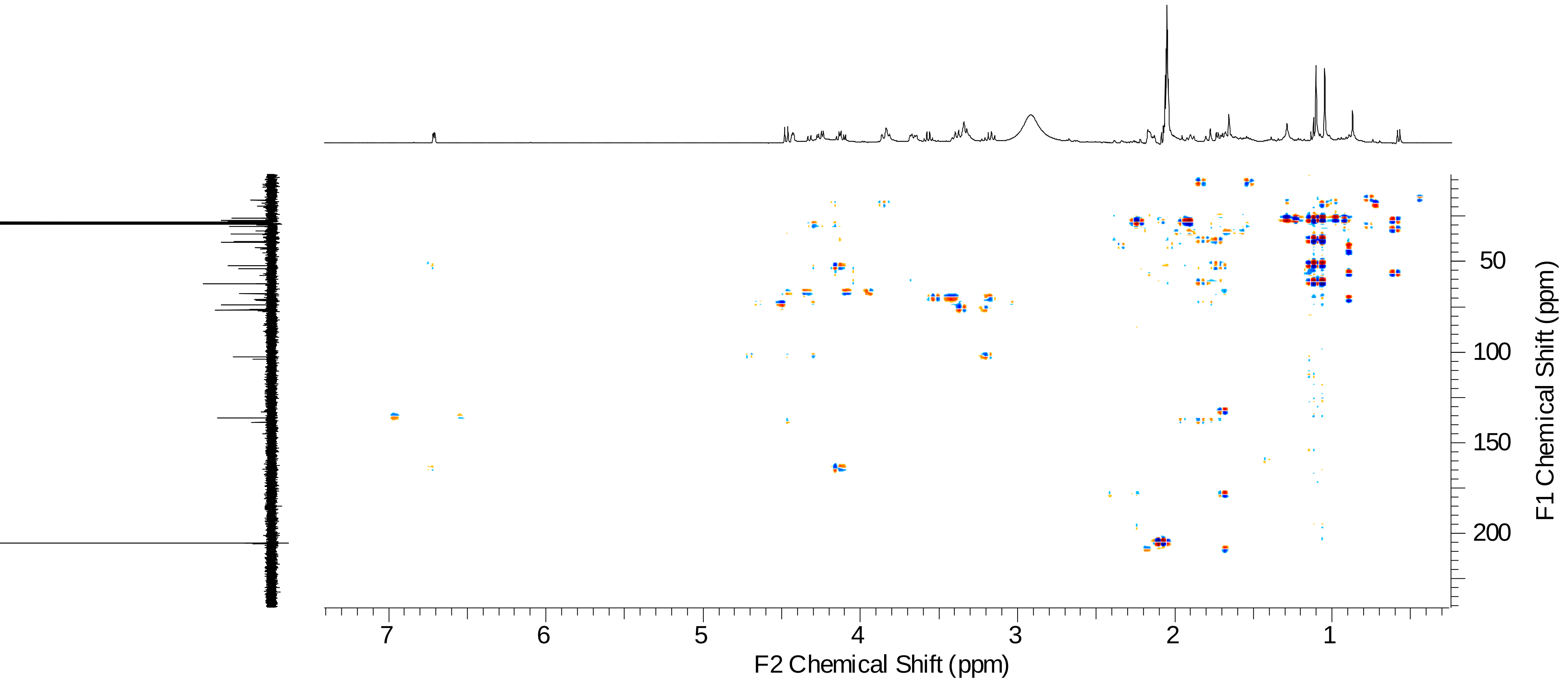

13 2D NMR NOESY correlation map of the sesquiterpene lactone **2** (400/400 MHz, acetone- d_6).

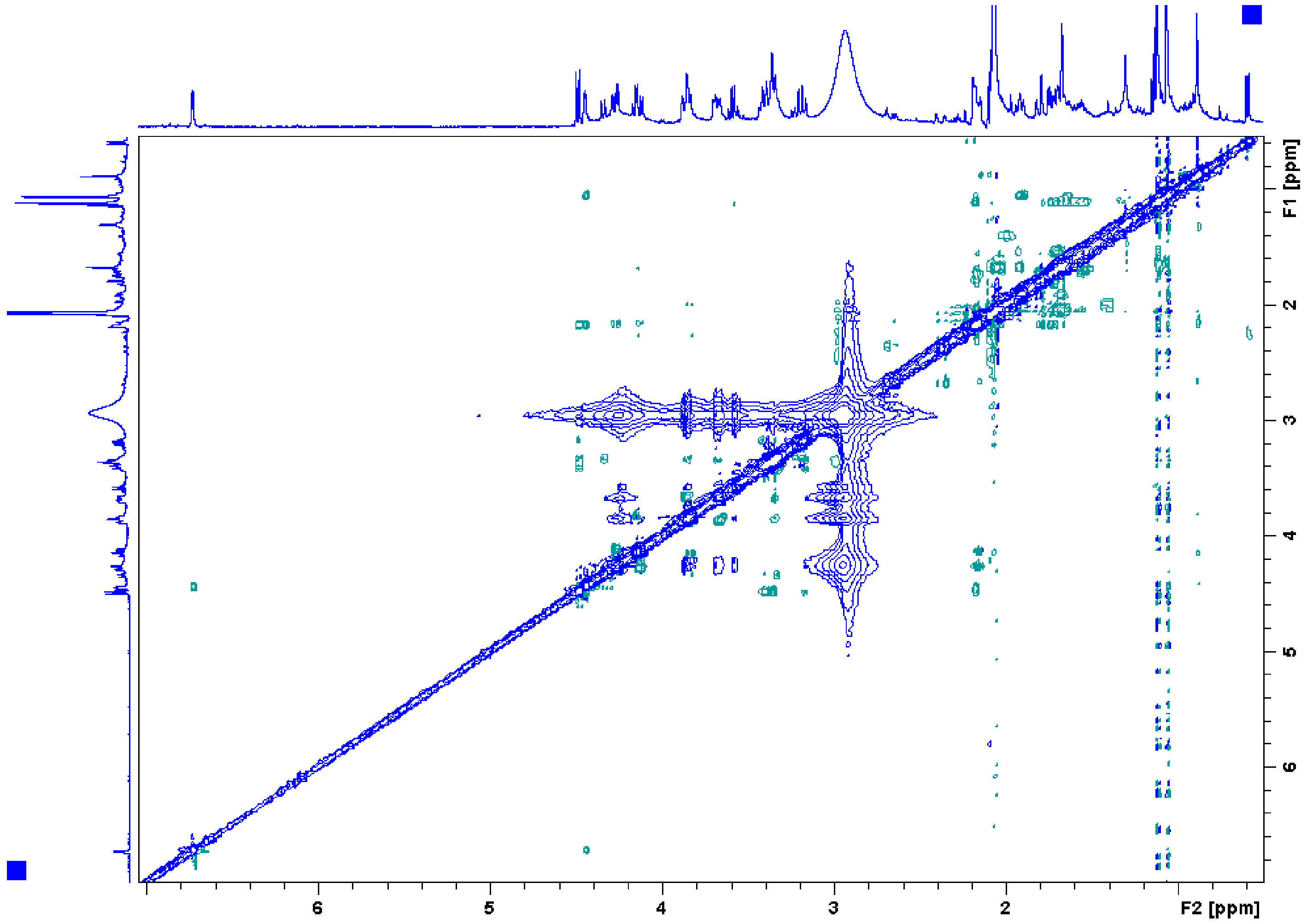

14 HR-MS spectrum of the sesquiterpene lactone **2**

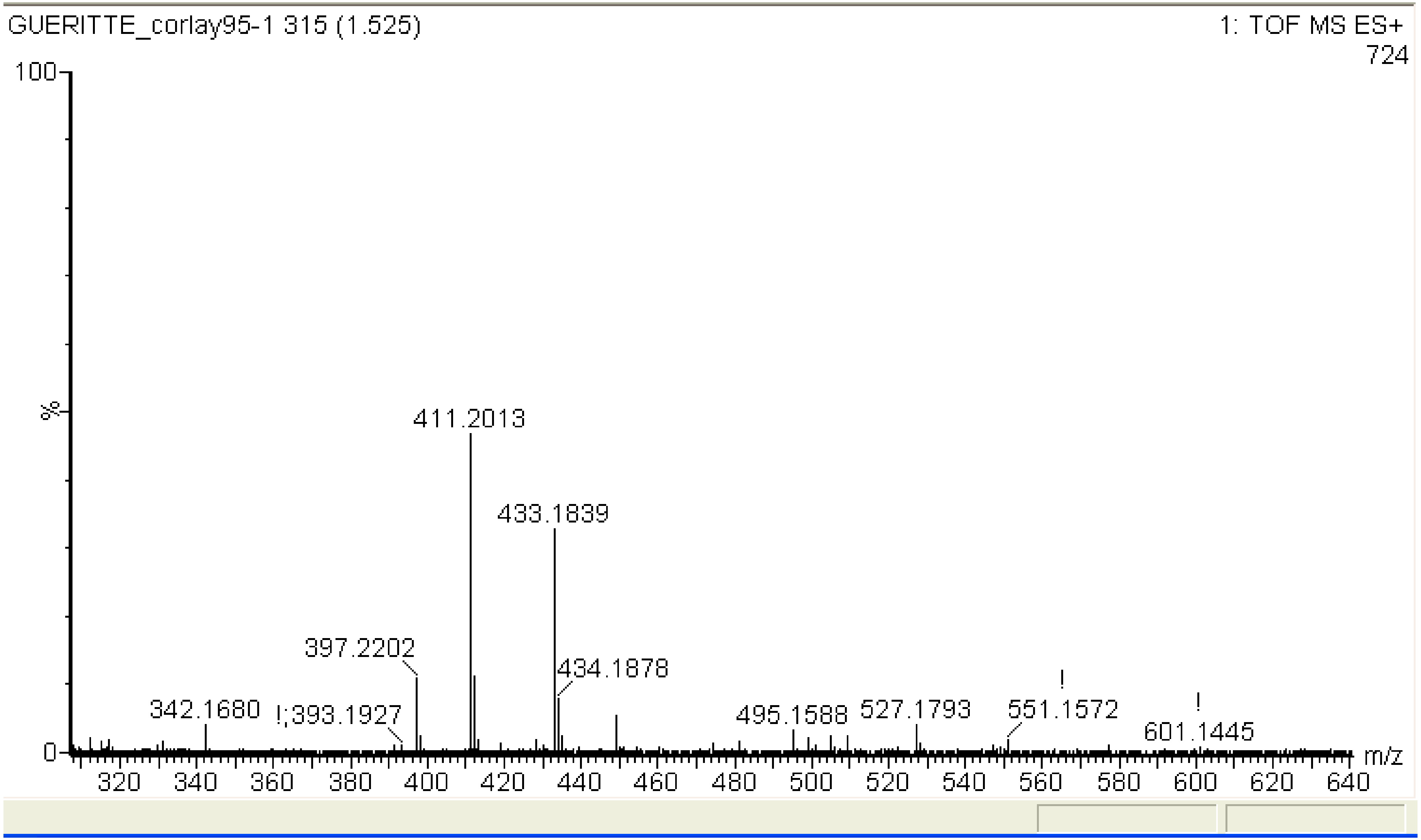

15 ^{1}H NMR spectrum of the sesquiterpene **3** (400 MHz, acetone- d_6).

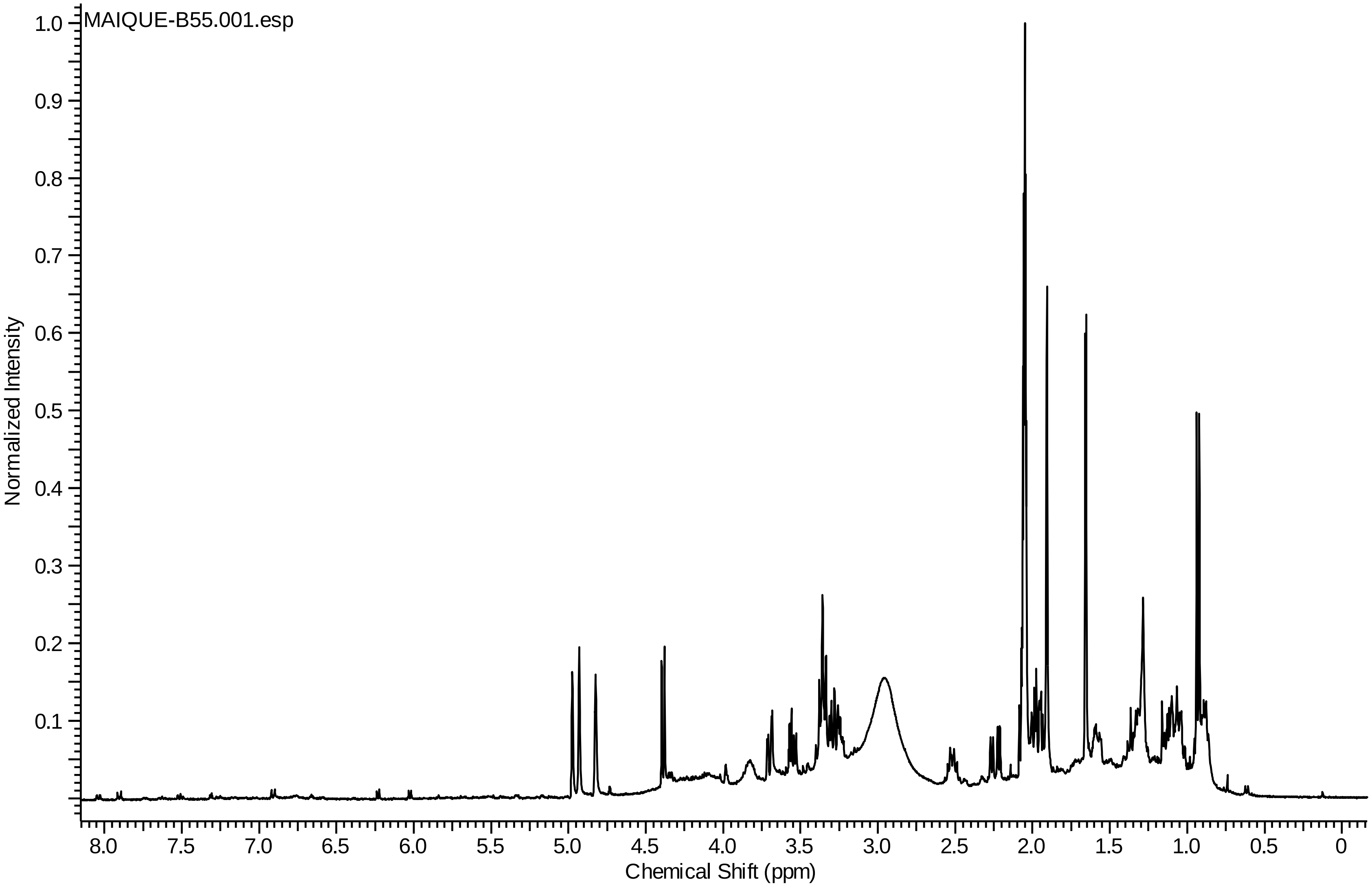

16 2D NMR HSQC correlation map of the sesquiterpene **3** (100/400 MHz, acetone- d_6).

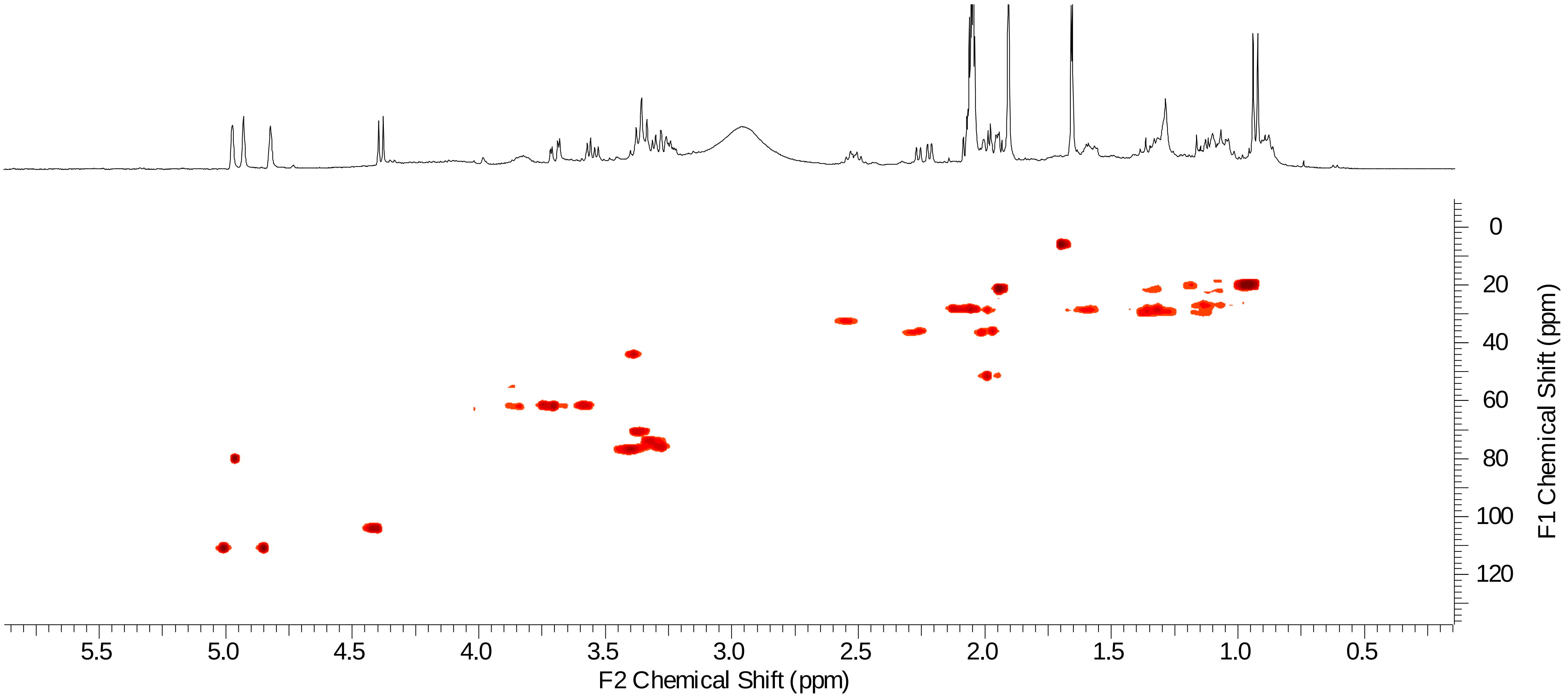

17 2D NMR COSY correlation map of the sesquiterpene **3** (400/400 MHz, acetone- d_6).

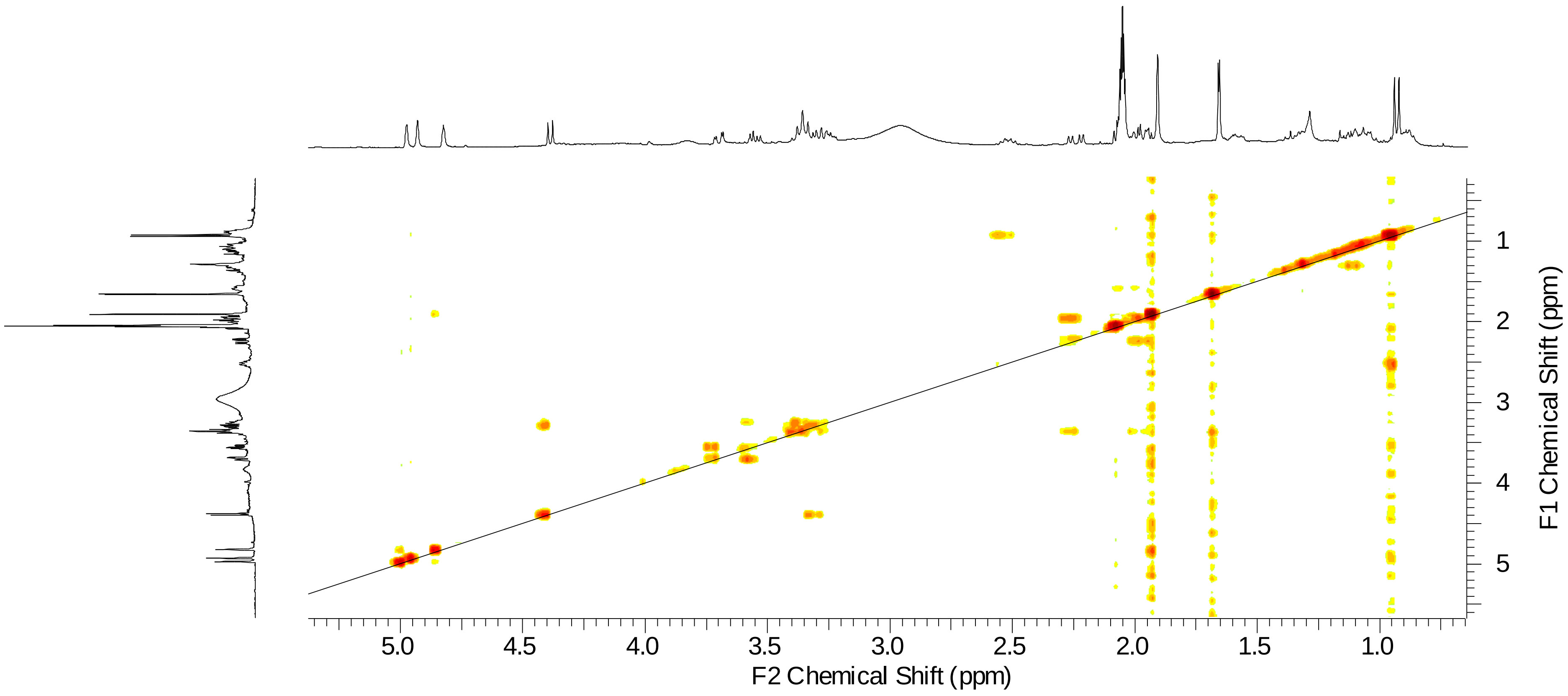

18 2D NMR HMBC correlation map of the sesquiterpene **3** (100/400 MHz, acetone- d_6).

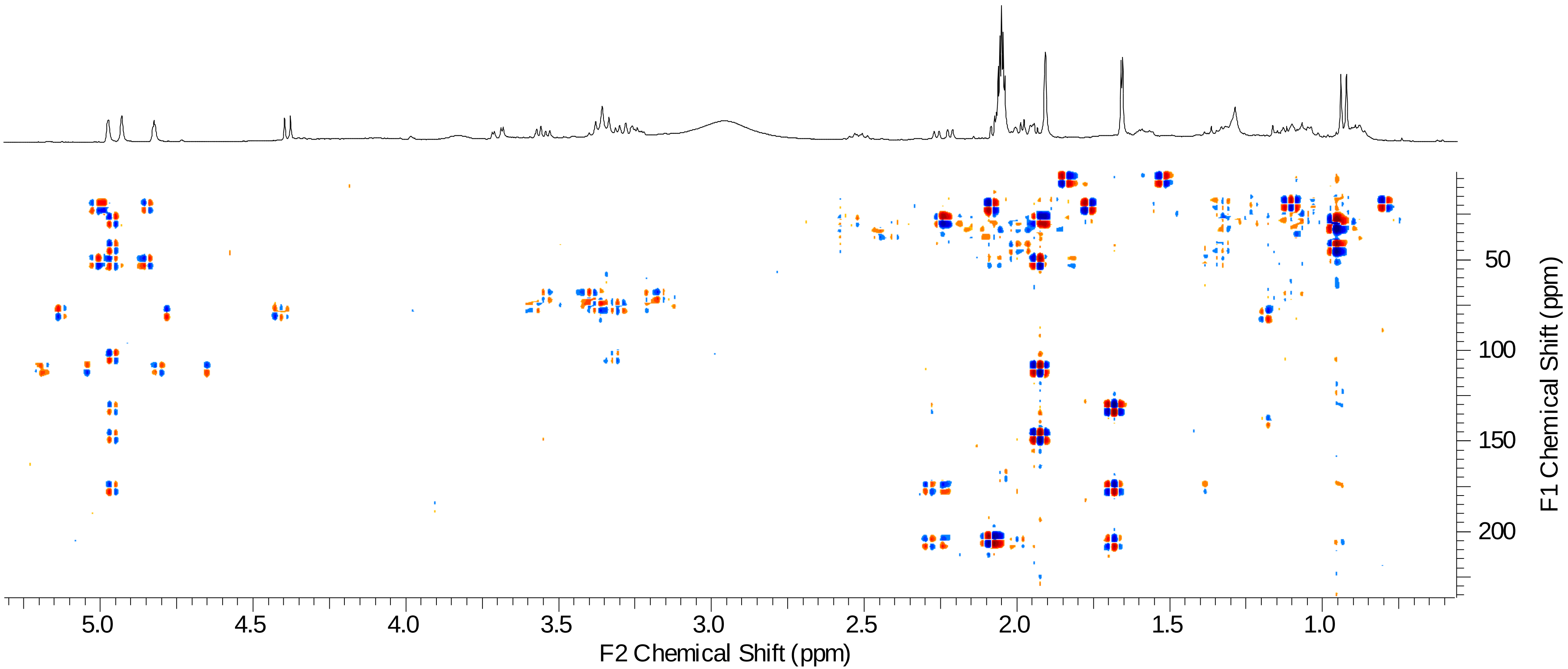

19 2D NMR NOESY correlation map of the sesquiterpene **3** (400/400 MHz, acetone- d_6).

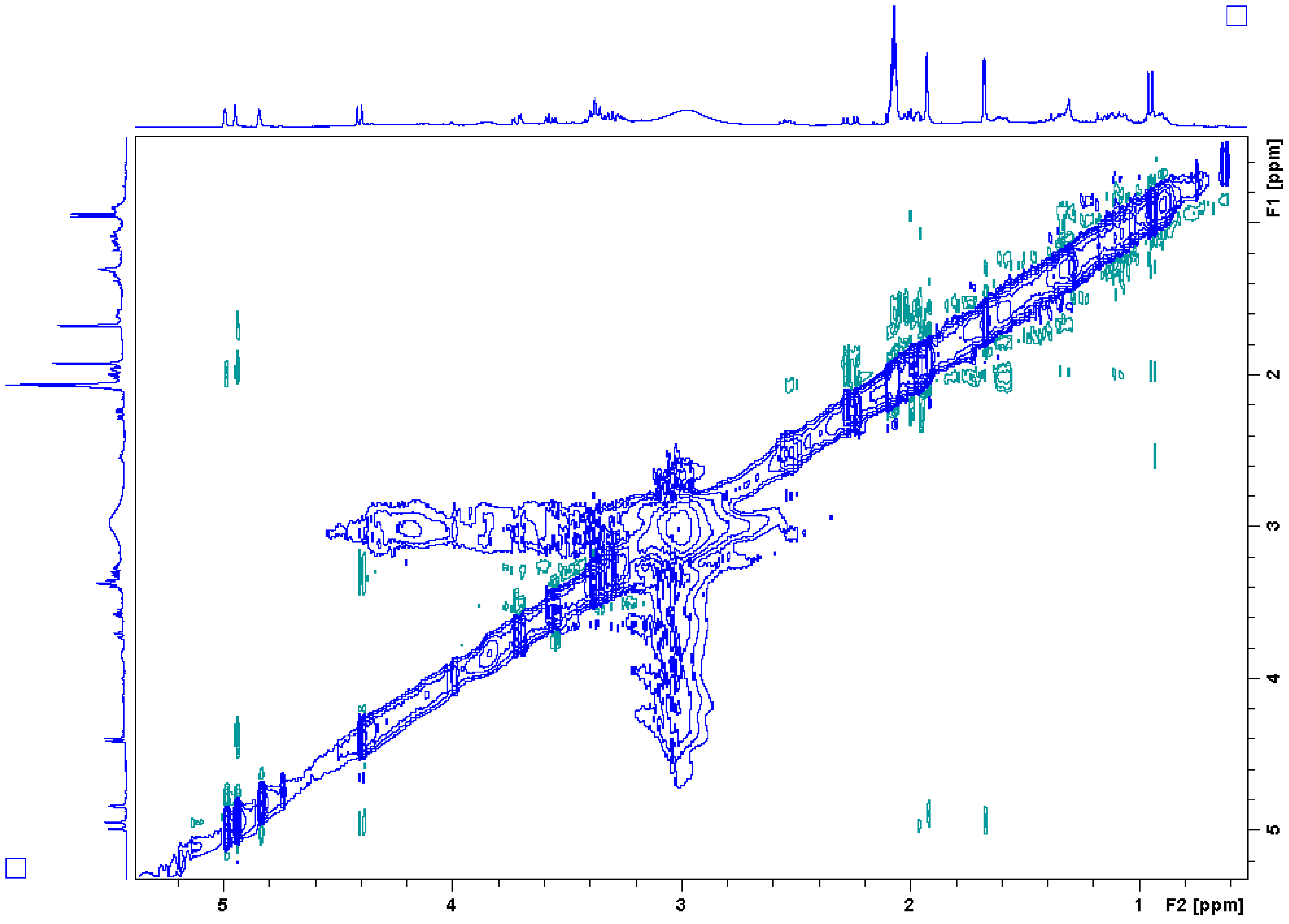

20 HR-MS spectrum of the sesquiterpene **3**

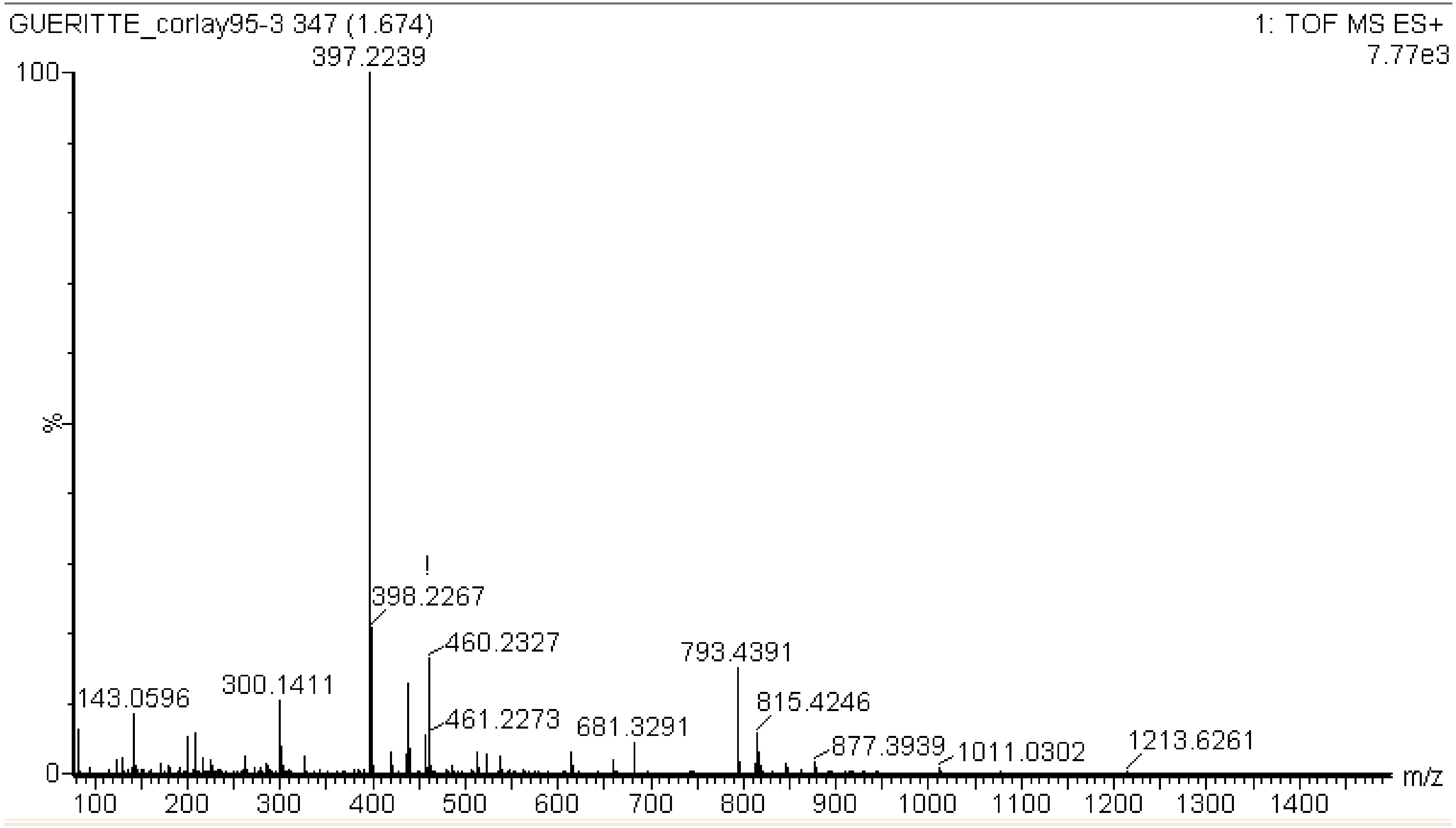

Supplement: File 1 — NMR and MS spectra of compounds 1–3. [file Beilstein_J_Org_Chem-12-674-s001.pdf]
